# PE_PGRS31-S100A9 Interaction Promotes Mycobacterial Survival in Macrophages Through the Regulation of NF-κB-TNF-α Signaling and Arachidonic Acid Metabolism

**DOI:** 10.3389/fmicb.2020.00845

**Published:** 2020-05-08

**Authors:** Sheng Liu, Yan Xie, Wei Luo, Yafeng Dou, Huan Xiong, Zhen Xiao, Xiao-Lian Zhang

**Affiliations:** ^1^Hubei Province Key Laboratory of Allergy and Immunology, Department of Immunology, Wuhan University School of Basic Medical Sciences and Department of Allergy, Zhongnan Hospital, Wuhan University, Wuhan, China; ^2^State Key Laboratory of Virology, Frontier Science Center for Immunology and Metabolism, Wuhan University School of Medicine, Wuhan, China

**Keywords:** *Mycobacterium tuberculosis*, Rv1768/PE_PGRS31, S100A9, tumor necrosis factor α (TNF-α), region of deletion (RD) 14, macrophage

## Abstract

*Mycobacterium tuberculosis* (*M. tb*) evades the surveillance of immune responses for survival in macrophages. However, the precise mechanism and toxins/proteins encoded by *M. tb* involved in the bacterial escape remain elusive. The function of Rv1768 protein (also referred to as PE_PGRS31, belonging to the PE_PGRS family) encoded by the region of deletion 14 (RD-14) in the virulent *M. tb* H37Rv strain has not, to the best of our knowledge, been reported previously. Here, we found that Rv1768 remarkably promotes bacterial survival in macrophages. Compared to wild type (WT) H37Rv, the Rv1768 deficient strain (H37RvΔ1768) showed significantly decreased colony-forming units in the lungs, spleen, and liver of the murine *M. tb* infection model. The bacterial burdens of WT H37Rv in WT macrophages and C57BL/6 mice were significantly higher than those in S100A9 deficiency cells and mice, but there were no significant differences for H37RvΔRv1768. Rv1768 binds S100A9 with the proline-glutamic acid domain (PE domain) and blocks the interaction between S100A9 and Toll-like receptor 4 (TLR4), and suppresses TLR4-myeloid differentiation factor 88-nuclear factor-kappa B (NF-κB)-tumor necrosis factor α (TNF-α) signaling in macrophages. Interestingly, Rv1768 binding to S100A9 also disturbs the metabolism of arachidonic acid by activating 5-lipoxygenase, increasing lipotoxin A4, and down-regulating cyclooxygenase-2 and prostaglandin E2 expression, thus, promoting mycobacterial survival. Our results revealed that *M. tb* Rv1768 promotes mycobacterial survival in macrophages by regulating NF-κB-TNF-α signaling and arachidonic acid metabolism via S100A9. Disturbing the interaction between Rv1768 and S100A9 may be a potential therapeutic target for tuberculosis.

## Introduction

Tuberculosis (TB) is an infectious disease caused by *Mycobacterium tuberculosis* (*M. tb*). Approximately a quarter of the world’s population has a latent TB infection, thus, they are at risk of developing active TB during their lifetime (World Health Organization [WHO], 2018). The only available vaccine, Bacillus Calmette-Guérin (BCG), an attenuated strain of *Mycobacterium bovis*, only decreases childhood TB and provides minimal protection against adult lung TB (Fine, 1995; Izzo, 2017). *M. tb* infects macrophages and persists in human macrophages for a prolonged period of time by escaping the host immune defense system (Pieters, 2008). *M. tb* has evolved multiple mechanisms to interfere with a wide range of host cellular processes, such as the modulation of macrophage survival (Queval et al., 2017), the production of cytokines (Ravan et al., 2019), reactive oxygen and nitrogen species (Tiwari et al., 2018; Mehta and Singh, 2019), the blockage of phagosome maturation (Zulauf et al., 2018), microtubule-associated light chain 3-associated phagocytosis and autophagy (Simmons et al., 2018). Tremendous efforts have been made to understand how *M. tb* survives in macrophages. Despite such efforts, many questions remain to be answered regarding the molecular mechanism of TB and the toxin encoded by *M. tb*. Therefore, further identification of bacterial toxins involved in bacterial escape will be useful to provide novel targets for anti-TB drugs and vaccines.

Comparative genomic studies have identified > 100 open reading frames distributed among several *M. tb*-specific genomic regions of deletion (RD), designated RD1 to RD16. These regions are present in the virulent H37Rv strain, but are absent in the non-virulent *M. bovis* BCG strain, suggesting that these RD may encode potential virulent antigens for bacterial pathogenesis and, thus, may be suitable as biomarkers for the diagnosis or improvement of vaccine effectiveness (Behr et al., 1999). Among the biomarkers discovered, the 10 kDa culture filtrate protein and 6 kDa early secreted antigenic target, encoded by RD1, are the most widely used for TB clinical diagnosis. Besides RD1, only a few molecules [e.g., MPT64 encoded by RD2 (Nicolo et al., 2010) and Rv2645 encoded by RD3 (Luo et al., 2015)] have been characterized and analyzed thus far (Tang et al., 2014; Luo et al., 2018). Therefore, identifying additional functional RD-encoded proteins might enhance our understanding of the pathogenesis of virulent *M. tb*.

Here, we focus on the screening of potential effector proteins encoded by RD regions from the H37Rv strain, which enable bacterial survival in macrophages. Approximately 51 different RD-encoded proteins were expressed and screened, and we found that Rv1768 [also referred to as PE- polymorphic GC-rich sequence (PGRS)31] from RD14 of H37Rv promoted the greatest bacterial intracellular survival in macrophages. We further clarified the mechanisms underlying Rv1768’s promotion of bacterial survival and inhibitory effects on the host inflammatory innate responses during mycobacterial infection in macrophages and murine TB models. Our findings suggest that *M. tb* Rv1768 binds S100A9 with the proline-glutamic acid (PE) domain and promotes mycobacterial survival in macrophages by regulating TLR4-myeloid differentiation factor 88 (MyD88)-nuclear factor-kappa B (NF-κB)-tumor necrosis factor-α (TNF-α) signaling and arachidonic acid metabolism. Our study provides insight into a therapeutic strategy based on blocking Rv1768 interaction with the host S100A9 to inhibit *M. tb* infection in macrophages.

## Materials and Methods

### Ethics Statement

All animal experimental protocols were performed in compliance with the Chinese National Laboratory Animal-Guideline for Ethical Review of Animal Welfare and were approved by the Institutional Animal Care and Use Committee of Wuhan University (No. 18021B) and the Second Military Medical University of Shanghai (No. 18002). All bacterial cultures and animal bacterial challenge experiments were carried out in the Animal Biosafety Level 3 Laboratories of the Wuhan University School of Medicine and the Second Military Medical University of Shanghai. The mice were euthanatized with CO_2_ prior to further examination.

### Animal Use

Female 6–8-week-old C57BL/6 mice were purchased from the Centre of Animal Experiments of Wuhan University. S100A9^–/–^ C57BL/6 mice were purchased from Cyagen Biosciences Inc. S100A9 KO mice harboring genetic deletion of the region exons 2 and 3 of mouse S100A9 were generated on the C57BL/6 background by Cyagen Biosciences using CRISPR/Cas9-based targeting and homology-directed repair. In the founder lines, the selected candidate genes were checked via PCR and sequencing to avoid off-target mutations. Heterozygous S100A9 mice were inter-crossed to generate homozygous targeted mice. SgRNAs and PCR amplification primers and the sequencing primers used are listed in Supplementary Table S1. TLR4 knockout (KO) and MyD88 KO C57BL/6 mice were kindly provided by Professors Hongliang Li (Wuhan University) and Zhinan Yin (College of Life Sciences, Nankai University), respectively.

### Bacterial Strains

*Mycobacterium tuberculosis* H37Rv (strain ATCC 27294), *M. tb* H37Ra (strain ATCC 25177), *M. bovis* BCG (Pasteur strain ATCC 35734), *Mycolicibacterium smegmatis* (*Ms*, strain ATCC 19420), *Mycobacterium marinum* (strain ATCC 927), *Mycobacterium intracellulare* (strain ATCC 13950), *Mycobacterium avium* (strain ATCC 25291), *Escherichia coli* DH5α (strain ATCC 25922), and *E. coli* BL21 (strain ATCC BAA-1025) were used in this study (Tang et al., 2016). *E. coli* DH5α, BL21, and *Ms* were grown in flasks using LB medium or 2 × YT medium. The mycobacterial strains were grown in Middlebrook 7H9 broth (271310; BD Difco) supplemented with 10% oleic acid-albumin-dextrose-catalase (OADC) and 0.05% Tween 80 (Sigma-Aldrich), or on Middlebrook 7H10 agar (262710; BD Difco) supplemented with 10% OADC and glycerol (5 mL in 1 L medium).

### Plasmids, Antibodies (Abs), and Reagents

The Rv1768 gene was amplified from the H37Rv genome and cloned into pET28a, pcDNA3.1-Myc-His, and pEGFP-C1, respectively. The mycobacterial shuttle vector pMV261 was used to express Rv1768 in *Ms*. The pSilencer1.0-U6 vector (Ambion) was used to construct S100A8 shRNA expression vectors in this study. Both pGL3-NF-κB-luc and Renilla were used in the Dual-Luciferase^®^ Reporter Assay System (Promega). The cDNAs of S100A9 and S100A8 were cloned into pAsRed2-N1. The following Abs were used in this study: anti-p-IκBα (2859s), anti-p-p65 (#3033), anti-5-LO (3289S), and anti-Cox-2 (#12282) from Cell Signaling Technology (Danvers, MA, United States); anti-S100A9 (DF7596) and anti-S100A8 (Abs136076) from Absin Bioscience Inc. (Shanghai, China); anti-GAPDH (WH086045) and anti-β-actin (WH090708) from ABclonal Biotechnology (Boston, United States), and anti-TLR4 (GTX75742) and anti-LAMP1 (ab24170) from GeneTex (San Antonio, United States) and Abcam (Cambridge, United Kingdom), respectively. Anti-Rv1768, anti-Rv3873, and anti-Rv1773 were rabbit polyclonal Abs prepared by our laboratory. Lipopolysaccharide (LPS) and Ni-NTA resin were purchased from Sigma-Aldrich (St. Louis, MO, United States) and Qiagen (Dusseldorf, German), respectively. Mouse macrophage colony stimulating factor was obtained from PeproTech (Rocky Hill, United States). Zileuton was purchased from Selleck (Houston, TX, United States). BAY11-7082 was obtained from MCE (Monmouth Junction, United States). Liposomes and clodronate liposomes (CL) were purchased from FormuMax (Sunnyvale, CA, United States).

### Bone-Marrow-Derived Macrophage (BMDM) Preparation

To prepare BMDMs, bone marrow cells isolated from mouse femoral bones were treated with erythrocyte-lysing buffer to remove red blood cells. The resultant cells were cultured in DMEM supplemented with 10% FBS, 1% antibiotic/antimycotic, and 50 ng/mL macrophage colony-stimulating factor (Peprotech, Rocky Hill, NJ, United States) for 6 days to induce differentiation into M0 macrophages. The purity of the F4/80^+^ BMDMs was assessed by flow cytometry.

### Cell Culture and Transfection

The murine RAW264.7 macrophage cell line (ATCC TIB-71) and S100A9^–/–^ RAW264.7 were cultured in DMEM (Gibco, United States) supplemented with 10% (v/v) FBS (NATOCOR, Cordoba, Argentina). Briefly, a targeting construct was produced through CRISPR/Cas9 to disrupt S100A9 in the RAW264.7 cell line by Jiangsu Genloci Biotechnologies Inc., China. The complementary oligonucleotide guide RNAs for S100A9 (listed in Supplementary Table S1) were annealed and cloned into pGK1.1 (Puro)/CRISPR Cas9. The recombinant plasmids were transfected into RAW264.7 cells using the SF Cell Line 4D-Nucleofector^®^ X Kit L (Lonza, United States). Cell transfection was performed with NEOFECT^TM^ DNA transfection reagent (Neofect Biotech Co., Ltd., Beijing), according to the manufacturer’s instructions. RAW264.7-TNF-α (TNF-α stably expressing cell line) was kindly provided by Professor Zhuoya Li from the Department of Immunology, Tongji Medical College, Huazhong University of Science and Technology, China.

### Construction of *Ms* Recombinant Strain

The shuttle expression plasmid pMV261-Rv1768 was electro-transformed into *Ms* using Gene Pulser Xcell^TM^ (Bio-Rad, United States) in 2 × YT medium with the following parameters: Voltage 2500 V, Capacitance 25 μF, Resistance 1500 Ω, and Cuvette 4 mm. Then, the generated recombinant *Ms* strains were selected by kanamycin on 2 × YT agar plates. Successful construction of *Ms* (pMV-1768) was confirmed by PCR with Rv1768 primers (Supplementary Table S1) and western blotting with rabbit anti-Rv1768 polyclonal Abs prepared in the lab.

### Mixed Infection Assay

Bacterial adherence and invasion assays were performed as previously described (Zhang et al., 2000). Briefly, for *Ms* strains, fresh wild type (WT) and S100A9^–/–^ RAW264.7/BMDMs (1 × 10^6^ cells) were seeded in six-well plates and mixed with bacteria (1 × 10^7^ colony forming units (CFUs) of *Ms* (pMV-1768), *Ms* (pMV), or *Ms*) at a ratio of 1:10 (eukaryotic to bacterial cells). For the H37Rv strain, fresh WT or S100A9^–/–^ RAW264.7/BMDMs (5 × 10^5^ cells) were seeded in 12-well plates and mixed with bacteria (5 × 10^6^ CFUs of H37Rv or H37RvΔ1768) at a ratio of 1:10. The mixtures were incubated at 37°C in a 5% (vol/vol) CO_2_ atmosphere for 1 h. Bacteria that did not enter the eukaryotic cells were removed with three washes of PBS and eliminated by incubation with gentamicin (200 μg/mL) for 3 h, followed by washing twice with PBS. Intracellular bacteria were recovered by eukaryotic cell lysis with 0.01% (wt/vol) Triton X-100 for 5 min and enumerated on 2 × YT medium agar plates supplemented with kanamycin or 7H10 agar plates supplemented with hygromycin. All experiments were performed at least three times.

### Ni-NAT Pull-Down and High-Performance Liquid Chromatography-Tandem Mass Spectrometry (MS) Analysis

To identify potential Rv1768-binding proteins in macrophages, Ni-NTA pull-down assay with Rv1768-His-Ni-NTA agarose followed by HPLC-tandem MS analysis were performed according to the methods of previous studies (Arnau et al., 2006). Recombinant Rv1768-6 × His-protein and Rv1768-6 × His-Ni resin were prepared. RAW264.7 cell lysates were incubated with Rv1768-6 × His-Ni-NTA resin or Ni-NTA agarose with shaking for 8 h at 4°C. His-Ni-agarose was washed three times using 10 mM imidazole solution. This was followed by the addition of 50 μL SDS loading buffer, boiling at 100°C for 10 min, and analysis by SDS-PAGE electrophoresis and Coomassie bright blue staining. The differential protein bands were collected and subjected to HPLC-MS/MS analysis (TripleTOF 5600plus, SCIEX, United States).

### Construction of the Rv1768 Deficient *M. tb* Strain H37RvΔ1768

Genetic manipulations of mycobacterial species were performed as described previously (Bardarov et al., 2002; Lee et al., 2004). Briefly, the H37Rv Δ1768 strain was constructed by allelic exchange using specialized transduction. The allelic exchange plasmids were constructed by amplifying 1 kb regions flanking Rv1768 with primers listed in Supplementary Table S1. Purified DNA fragments were digested with the *Van*91I restriction enzyme. Fragments were ligated with *Van*91I-digested p0004s (courtesy of Prof. T. Hsu, unpublished data) using T4 DNA ligase (NEB). The resulting recombinant plasmid was digested with *Pac*I and ligated to the *Pac*I-digested phAE159 shuttle plasmid. After ligation, the resulting cosmid phAE159-p0004s was transduced into *E. coli* HB101 for packaging into phasmids using MaxPlax packaging extract (Epicenter Biotechnologies). The phasmids were transformed into *Ms* by electroporation for phage propagation. The transducing phage was used to infect H37Rv at an MOI of 10. Successful specialized transduction was confirmed by PCR using primers listed in Supplementary Table S1 and western blot analysis.

### Mycobacterial Subcellular Protein Fractionation

*Mycobacterium tuberculosis* H37Rv was grown in 50 mL of 7H9 medium until mid-log phage and harvested by centrifugation at 3000 × *g* for 15 min at 4°C. The subcellular fraction was isolated by ultracentrifugation (Delogu et al., 2004; Cascioferro et al., 2007). Briefly, bacterial cells were resuspended in 10 mL PBS with 1 mM phenylmethane sulfonyl fluoride, and the samples were sonicated (Vibra cellTM Sonic&Materials Inc, United States) in an ice bath for 5 min. The lysates were centrifuged at 11,000 × *g* for 5 min at 4°C to precipitate cellular debris. The resulting supernatants were extracted by centrifugation at 27,000 × *g* at 4°C for 30 min to collect cell wall (CW)-associated proteins. Next, the supernatant was precipitated by ultracentrifugation at 110,000 × *g* for 2 h, and the cytoplasmic membrane (pellet) was separated from the cytosolic fraction (supernatant). Cytosolic proteins were subsequently precipitated by incubation with 10% trichloroacetic acid (v/v) on ice for 30 min and centrifugation for 10 min at 16,000 × *g* at 4°C, followed by washing with 80% acetone (v/v). The subcellular fractions obtained were analyzed by western blotting using anti-Rv1768, anti-Rv3873, and anti-Rv1773 rabbit polyclonal Abs prepared in the lab.

### Proteinase K Sensitivity Assay

Bacterial cells (8 × 10^9^ CFUs H37Rv) were washed with TBS buffer (Tris-HCl pH 7.5, NaCl 150 mM, KCl 3 mM) treated with or without 100 mg/mL proteinase K (Sigma-Aldrich) and incubated at 4°C for 60 min (Cascioferro et al., 2007). The reaction was stopped by adding a complete EDTA-free inhibitor (Roche). Samples were washed once in TBS, re-suspended, and subjected to SDS-PAGE and western blot analysis with anti-Rv1768, anti-Rv3873, and anti-Rv1773 rabbit polyclonal Abs, respectively.

### Immunoprecipitation (IP) and Immunoblot Analyses

We analyzed the interactions between Rv1768/PE-domain/PGRS-domain and Sl00A9/S100A8/TLR4 as follows: the cell lysates of WT RAW264.7 cells were incubated with Rv1768/PE-domain/PGRS-domain-His-Ni-NTA-agarose overnight. The pull-down products were subjected to western blot analysis with anti-Rv1768, anti-S100A8/9, or anti-TLR4.

To analyze the effects of Rv1768 on the interactions between S100A9 and TLR4, RAW264.7 cells were transfected with pcDNA3.1 or pcDNA3.1-RV1768. At 24 h post-transfection, cell lysates were incubated overnight with anti-S100A9 and 30 μL Protein A/G Magnetic Beads (MCE, Monmouth Junction, United States) at 4°C in a low-speed horizontal shaker. The immune-precipitation protein A/G-bead complex was centrifuged at 3,000 × *g*, 4°C for 5 min, and the pellets were washed with 1 mL of lysis buffer three times, followed by western blot analysis with anti-Rv1768, anti-S100A9, or anti-TLR4. The transient transfection efficiency of the RAW264.7 cells was measured using enhanced GFP as a reporter molecule. The percentages of transfected cells were determined using pEGFP-C1 plasmid transfected RAW26.7 cells. Histogram quality and the percentage of transfected cells were determined (based on the number of fluorescent cells counted) using flow cytometer. A transfection efficiency of approximately 35.8–40% was observed for GFP-positive cells.

### RT-qPCR

Total RNA was extracted using TRIzol reagent (Invitrogen, Carlsbad, CA, United States). First-strand cDNA was synthesized from total RNA using the ReverTra Ace-First strand cDNA Synthesis Kit (Toyobo, Osaka, Japan), according to the manufacturer’s instructions. RT-qPCR was conducted in a 96-well microtiter plate with a SYBR Green real-time PCR MasterMix kit (Toyobo, Osaka, Japan) on an ABI Step One Plus^TM^ Real-Time PCR system (Applied Biosystems). GAPDH mRNA was used as a reference housekeeping gene for normalization. The fold-change was calculated with 2^–^^Δ^^Δ^^*Ct*^, where ΔΔCt = experimental group Ct (Ct _*target*_-Ct _*GAPDH*_)-control group Ct (Ct _*target*_- Ct _*GAPDH*_). All experiments were performed at least three times.

### Confocal Microscopy Analysis

To analyze the co-localization of Rv1768 and S100A8/9, RAW264.7 (3 × 10^5^) cells were seeded in confocal dishes (NEST Biological Technology Co., Ltd., Shanghai, China) and co-transfected with pEGFP-C1-PE/pEGFP-C1-PGRS/pEGFP- C1-Rv1768 and pAsRed2-N1-S100A9/pAsRed2-N1-S100A8. Then, 24 post-transfection, the nuclei were labeled with DAPI. Confocal images were taken with a Leica-LCS-SP8-STED confocal system.

### Enzyme-Linked Immunosorbent Assay (ELISA)

WT or S100A9^–/–^ BMDMs/RAW264.7 cells were infected with H37Rv or H37RvΔ1768 at different time points (0, 0.5, 1, 2, 4, 6, and 8 h), and supernatants were collected to detect TNF-α, interleukin 6 (IL-6), and IL-1β expression using ELISA kits (Dakewe Biotech, Beijing, China), according to the manufacturer’s instructions. Both WT and S100A9^–/–^ C57BL/6 mice were infected with H37Rv or H37RvΔ1768. On day 28 post-infection, mouse sera were isolated for the detection of TNF-α by ELISA kits (Dakewe Biotech, Beijing, China), according to the manufacturer’s instructions.

Both WT and S100A9^–/–^ RAW264.7 cells were infected with H37Rv or H37RvΔ1768 at different time points (0, 4, 8, 12, 16, 20, and 24 h). The supernatants were collected to detect the levels of LTB4, lipotoxin A4 (LXA4), and prostaglandin E2 (PGE2) using ELISA kits (R&D Systems, Minneapolis, United States), according to the manufacturer’s instructions.

### Flow Cytometric Analysis

To assess the purity of murine BMDMs, briefly, the isolated cells were filtrated, then stained with 0.25 μg APC-anti-mouse F4/80 Ab (Biolegend, San Diego, CA, United States), per 1 × 10^6^ cells at 4°C for 30 min in the dark. Then, the cells were washed twice with PBS and analyzed by flow cytometry with a Beckman CytoFLEX FCM (CA, United States).

### Dual Luciferase Reporter Assay

A dual luciferase reporter assay was performed as described previously (Li et al., 2007). The Dual-Luciferase^®^ Reporter Assay System (Promega, Madison, United States) was used to detect NF-κB activation using a GloMax^®^ 20/20 tube luminometer (Promega, United States). Briefly, RAW264.7 cells were co-transfected with pGL3-NF-κB-luc, Renilla, pcDNA3.1-Rv1768, or pcDNA3.1. Then, 24 h post-transfection, cells were treated with LPS (1000 ng/mL, Sigma-Aldrich) to stimulate NF-κB activation. After 1 h, the cells were collected. Luciferase activity was measured using the Dual-Luciferase Reporter Assay System according to the manufacturer’s instructions (Promega). Data were normalized for transfection efficiency by dividing firefly luciferase activity with that of Renilla luciferase.

### Mouse Infection Experiments

For the mouse infection experiments, both WT and S100A9^–/–^ C57BL/6 mice (pre-treated with isoflurane for anesthesia) were intranasally (*i.n.*) infected with 50 μL (1 × 10^6^ CFU/mL, 5 × 10^4^ CFUs per mouse) of H37Rv or H37RvΔ1768 strains (Leemans et al., 2003; Wieland et al., 2008; Lee et al., 2017). The bacteria were dispersed into single cell suspensions by sonication with BACspreader^TM^ 1100 (TB Healthcare, China) before infection. The bacterial numbers were determined by viable counts on Middlebrook 7H10 agar plates. On days 7 and 28 post-infection, the bacterial numbers in the organs of infected mice were assessed by plating organ homogenates onto agar plates and enumerating CFUs after 3 weeks of culturing. Mouse sera were collected on day 28 post-infection for cytokine measurements by ELISA. The lung tissues obtained on day 28 post-infection were subjected to fast-acid staining with hematoxylin and eosin and Ziehl–Neelsen acid-fast staining.

For mouse adoptive transfer experiments (Kaufmann et al., 2018), WT C57BL/6 mice (pre-treated with isoflurane for anesthesia) were intravenously (*i.v.*) injected with CLs or liposome controls (200 μL per mouse) to deplete macrophages. The depletion efficiency was evaluated by flow cytometry analysis. On days 2 and 5 post-injection, mice were adoptively transferred with BMDMs (5 × 10^6^ cells per mouse) derived from WT or S100A9^–/–^ C57BL/6 mice. Five days after the first adoptive transfer, the mice were challenged with 1 × 10^8^ CFUs of *Ms* (pMV) or *Ms* (pMV-1768). One day after infection the mice were euthanized, and the concentrations of serum TNF-α and bacterial CFUs were determined.

### Statistical Analysis

Data are presented as the mean ± SD and were analyzed using GraphPad Prism Version 8.0 software (GraphPad Software, San Diego CA, United States). Differences between groups were tested using an unpaired Student’s *t*-test (two-tailed). One- or two-way analysis of variance (ANOVA) with Tukey’s *post hoc* multiple comparison test were used to compare the means across multiple groups. Two-way repeated measures ANOVA with Tukey’s *post hoc* multiple comparison test were used to compare the means across multiple time points and groups. All data met the assumptions of the statistical tests. Two-sided *p* values of less than 0.05 were considered statistically significant (*p < 0.05, **p < 0.01, ***p < 0.001, ****p < 0.0001).

## Results

### *M. tb* RD14-Encoded Rv1768 Significantly Promotes Bacterial Invasion and Survival in Macrophages

To identify H37Rv RD-encoded candidate proteins that are capable of promoting bacterial survival in macrophages, 51 RD-encoded proteins were cloned into the pET28a vector and expressed in *E. coli* BL21 by IPTG induction. These 51 *E. coli* BL21 strains carrying pET-28a-RDs were incubated with fresh mouse peritoneal macrophages (MOI = 10) for 1 h. Bacteria that did not enter the eukaryotic cells were removed by three washes with PBS and eliminated by incubation with gentamicin, followed by washing twice with PBS. The bacterial invasion assay showed that the *E. coli* BL21 strain carrying Rv1768 (pET-28a-Rv1768) had the highest bacterial CFU on LB plates compared to the *E. coli* BL21 strains carrying other RD-encoded genes (data not shown).

**FIGURE 1:**
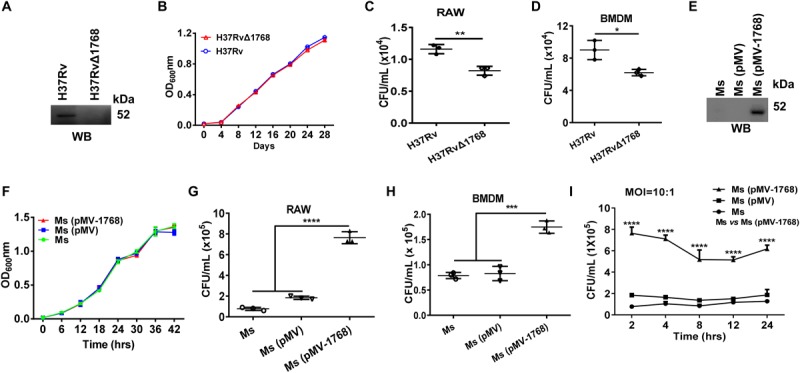
Rv1768 promotes bacterial invasion and survival of macrophages. **(A)** Western blot analysis of the lysates of WT H37Rv strain and Rv1768 deletion strain (H37RvΔ1768) using anti-Rv1768 Ab**. (B)** Growth curves of WT H37Rv and H37RvΔ1768 strains**. (C,D)** RAW264.7 cells **(C)** or murine BMDMs (5 × 10^5^) **(D)** were infected with 5 × 10^6^ CFUs WT H37Rv or H37RvΔ1768. After removal of bacteria that did not enter the eukaryotic cells, intracellular bacteria were recovered by eukaryotic cell lysis and enumerated. Unpaired Student’s *t*-test was used to compare the means between two groups. **(E)** Western blot analysis of the lysates of *M. smegmatis* (*Ms*), *M. smegmatis* transfected with pMV261 [*Ms* (pMV)] and *M. smegmatis* transfected with pMV261-Rv1768 [*Ms* (pMV-1768)] using anti-Rv1768 Ab**. (F)** Growth curve of *Ms*, *Ms* (pMV) and *Ms* (pMV-1768). **(G,H)** RAW264.7 cells **(G)** or murine BMDMs **(H)** (1 × 10^6^) were infected with 1 × 10^7^ CFUs *Ms*, *Ms* (pMV) and *Ms* (pMV-1768), for mixed infection assay as described in Materials and Methods. One-way analysis of variance (ANOVA) with Tukey’s multiple comparison test was used to compare the means across multiple groups. **(I)** RAW264.7 cells were infected with *Ms*, *Ms* (pMV) and *Ms* (pMV-1768) for different time (2, 4, 8, 12, 24 h) for mixed infection assay. Two-way repeated measures ANOVA with Tukey’s *post hoc* multiple comparison test was used to compare the means across multiple time points and multiple groups. The data are presented as mean ± SD (error bars) (*n* = 3). Data averaged from at least three independent experiments. *p < 0.05, **p < 0.01, ***p < 0.001, ****p < 0.0001.

To determine the function of Rv1768 in mycobacteria, we generated a novel Rv1768 deletion mutant strain-H37RvΔ1768 by allelic exchange using specialized transduction, as described in the “Methods and Materials” section (Supplementary Figures S1A–D). H37RvΔ1768 was confirmed by western blotting using anti-Rv1768 Ab (Figure 1A), and had a similar growth curve to that of the WT H37Rv strain (Figure 1B). Both WT H37Rv and H37RvΔ1768 strains were used to infect RAW264.7 cells and murine BMDMs (Supplementary Figure S1E) for invasion assays using mixed infections. Compared to the WT H37Rv strain, the H37RvΔ1768 strain displayed much lower bacterial CFUs in both RAW264.7 macrophages (**p < 0.01, Figure 1C) and BMDMs (*p < 0.05, Figure 1D). These results strongly suggest that Rv1768 promotes mycobacterial invasion and the survival of macrophages.

*Ms* is a rapidly growing bacterium (Gupta et al., 2018; Yamada et al., 2018; Nikitushkin et al., 2020) that is frequently used as a substitute for *M. tb* in studies (Doddam et al., 2019; Kaur and Kaur, 2019; Maslov et al., 2019; Wang et al., 2019). The full-length Rv1768 gene (1768 bp) was cloned into the mycobacterial shuttle vector pMV261 and transformed into *Ms* to construct a recombinant *Ms* expressing Rv1768 [*Ms* (pMV-1768)] (Supplementary Figures S1F,G). *Ms* (pMV-1768) and control strains [*Ms* (pMV) and *Ms*] were confirmed by western blotting (Figure 1E). Similar growth curves were observed between *Ms* (pMV-1768) and control strains (*Ms* (pMV) and *Ms*) (Figure 1F). These strains were used to infect RAW264.7 cells and BMDMs in the invasion assays. Our results showed that *Ms*(pMV-1768) had approximately four-fold higher bacterial CFUs in RAW264.7 cells (****p < 0.0001, Figure 1G) and two-fold higher bacterial CFUs in BMDMs (***p < 0.001, Figure 1H) compared to the control groups [*Ms*(pMV) and *Ms*]. These bacteria were also used to infect RAW264.7 cells (MOI = 10:1) for different time periods (2, 4, 8, 12, and 24 h). Compared to the control groups, *Ms* (pMV-1768) had about 3.5-fold more bacterial CFUs in RAW264.7 cells (****p < 0.0001, Figure 1I) at the indicated time points, suggesting that Rv1768 significantly promotes bacterial invasion and survival of macrophages.

### Rv1768 Is Associated With the Bacterial CW

**FIGURE 2:**
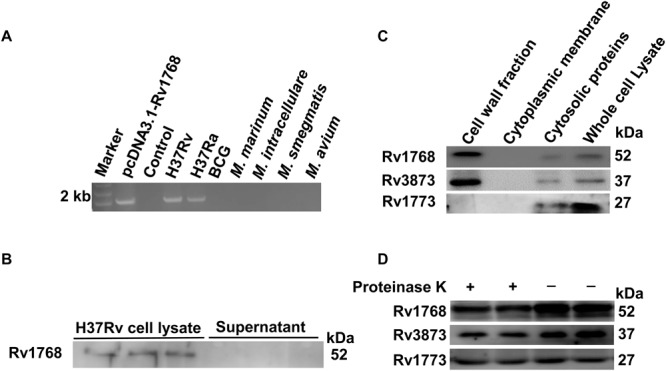
Rv1768 is associated with bacterial cell wall. **(A)** Agarose electrophoresis of Rv1768 PCR amplification products with genomic DNA of different mycobacteria species. **(B)** Western blot analysis of H37Rv cell lysate and supernatant with anti-Rv1768 antibody. **(C)** Western blot analysis of different mycobacterial subcellular protein fractionation with anti-Rv1768, anti-Rv3873, and anti-Rv1773, respectively. **(D)** Proteinase K sensitivity assay for H37Rv lysate after treatment with proteinase K.

Rv1768 belongs to the PE_PGRS family, which is characterized by an N-terminal PE domain and a C-terminal PGRS domain with a variable number of GGAGGN repeats (De Maio et al., 2014; Deng et al., 2017; Yang et al., 2017). Rv1768 is also referred to as PE_PGRS31, and the function of PE_PGRS31 has not yet been reported. We analyzed the Rv1768 gene distribution in different *Mycobacteriaceae* family strains by PCR and found that Rv1768 only exists in the H37Rv and H37Ra strains, but not in *M. marinum*, *M. intracellulare*, *M. avium*, *Ms*, or BCG strains (Figure 2A). As Rv1768 has a putative signal peptide at its N-terminal, we next explored whether Rv1768 is a secreted protein. However, we did not detect Rv1768 protein in the supernatants of H37Rv cultures (Figure 2B).

Several studies have shown that PE_PGRS proteins are located in the bacterial CW (Brennan et al., 2001; Chai et al., 2019). Therefore, we determined whether Rv1768 is located in the bacterial CW. We performed cell fractionation assays as described in the “Materials and Methods” section. Rv1768 protein was detected in the CW fraction (Figure 2C, left lane 1), but not in the cytoplasmic membrane section (Figure 2C, lane 2). Rv1768 is similar to the previously reported *M. tb* CW protein Rv3873 (Demangel et al., 2004) (Figure 2C), but different from the intracellular protein Rv1773 (Alexander and Behr, 2007) (Figure 2C). After further treatment with proteinase K for bacterial lysate, H37Rv bacterial CW proteins (both Rv1768 and Rv3873) were significantly degraded compared to cellular plasma proteins (such as Rv1773) (Figure 2D). These results suggest that the Rv1768 protein is located in the bacterial CW of the *M. tb* H37Rv strain.

### Rv1768 Promotes Mycobacterial Growth and Survival in Mice

**FIGURE 3:**
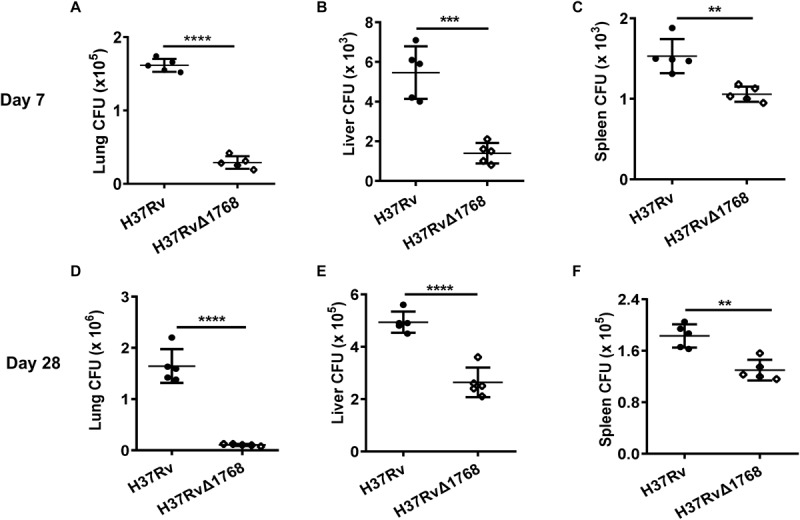
Rv1768 promotes mycobacteria invasion and survival in mice. Bacterial burden in the lungs **(A,D)**, liver **(B,E)**, and spleen **(C,F)** of mice infected (*i.n.*) with 5 × 10^4^ CFUs of H37Rv or H37RvΔ1768 (*n* = 5, each group) at 7 **(A–C)** or 28 Days **(D–F)** post infection. The data are presented as mean ± SD (error bars). Data averaged from at least two independent experiments. Unpaired Student’s *t*-test was used to compare the means between two groups (*vs.* H37Rv). *p < 0.05, **p < 0.01, ***p < 0.001, ****p < 0.0001.

Next, we examined whether Rv1768 could promote mycobacterial survival *in vivo*. C57BL/6 mice were infected *i.n.* with H37Rv or H37RvΔ1768 strains, according to a previous report (Santosuosso et al., 2007). H37Rv is a slow-growth bacterium. Compared to the H37RvΔ1768 group, we found that the H37Rv group had approximately three-fold more bacterial colonies in both the lungs (****p < 0.001, Figure 3A) and the liver (***p < 0.01, Figure 3B), and approximately 1.5-fold more in the spleen (**p < 0.01, Figure 3C) on day 7 post-infection. On day 28 post-infection, the H37Rv group had approximately 10-fold more bacterial colonies in the lungs (****p < 0.001, Figure 3D), two-fold more in the liver (****p < 0.001, Figure 3E), and 1.5-fold more in the spleen (**p < 0.01, Figure 3F), compared to the H37RvΔ1768 group. These results strongly suggest that Rv1768 significantly promotes mycobacterial survival in mice.

### Rv1768 Interacts With the S100A9/A8 Heterodimer

**FIGURE 4:**
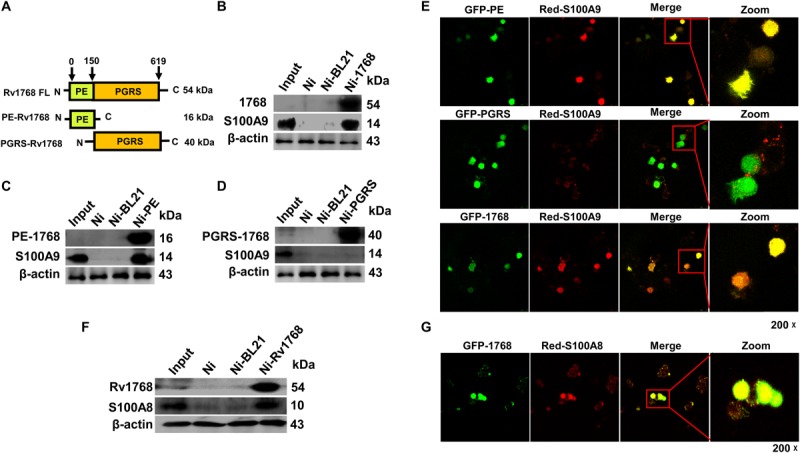
Rv1768 binds to S100A9/S100A8 of macrophage. **(A)** Diagram of full-length and truncated Rv1768 constructs. **(B–D)** Pull-down and immunoblot analysis of the interaction between Rv1768 **(B)**/PE domain **(C)**/PGRS domain **(D)** and S100A9. For pull down assay, bait proteins (Rv1768, PE domain of Rv1768, and PGRS domain of Rv1768) were immobilized onto Ni-NTA resin, followed by incubation with corresponding prey proteins in a RIPA buffer. After incubation and washing, the bound proteins were analyzed by immunoblotting analysis. **(E)** Confocal microscope analysis of the colocalization between Rv1768/1768-PE/1768-PGRS and S100A9 in RAW264.7 cell. RAW264.7 cells were co-transfected with pAsred2-N1-S100A9 and each of the three truncated or full-length protein plasmids (pEGFP-C1-PE, pEGFP-C1-PGRS, or pEGFP-C1-Rv1768). After 24 h, Confocal images were taken with a Leica-LCS-SP8-STED confocal system. The colocalization percentages were analyzed by ImageJ software. Manders’ M1 represents the overlap fraction of GFP fluorescence (green) in the area of AsRed2 fluorescence (red), and Manders’ M2 represents the overlap fraction of AsRed2 fluorescence (red) in the area of GFP fluorescence (green). For **(E)** upper panel, Manders’ M1 = 0.869 (86.9%); Manders’ M2 = 0.827 (82.7%). For **(E)** middle panel, Manders’ M1 = 0.489 (48.9%); M2 = 0.452 (45.2%). For **(E)** lower panel, Manders’ M1 = 0.714 (71.4%); M2, 0.706 (70.6%). **(F)** Western blot analysis of Rv1768-His-Ni-NTA agarose pull-down products of RAW264.7 cell lysates with anti-Rv1768 and anti-S100A8. **(G)** Confocal microscopy analysis of colocalization of Rv1768 and S100A8 in RAW264.7 cells co-transfected with pEGFP-Rv1768 and pAsRed2-N1-S100A8. For **(G)**, Manders’ M1 = 0.994 (99.4%); M2 = 0.847 (84.7%). Larger Manders’ M1 or M2 values indicate more co-localization.

To elucidate the mechanisms underlying Rv1768-mediated mycobacterial survival in macrophages, we searched for the host targets of Rv1768 protein in macrophages. The truncated or full-length Rv1768 expression plasmids in pET28a with His tags were constructed, including PE-1768 (PE domain, N-terminal domain of Rv1768), PGRS-1768 (PGRS domain, C-terminal domain of Rv1768), and full-length Rv1768 (Figure 4A), and were expressed and purified in *E. coli*. These proteins were conjugated to Ni-agarose and used to pull-down interaction proteins from RAW264.7 cell lysate, followed by SDS-PAGE and MS analysis (data listed in Supplementary Table S2). MS data analysis showed that PKM, S100A9, and TOP1 may be Rv1768-associated target proteins. We further used His-Ni-agarose pull-down and immunoblot analysis to confirm that only S100A9 directly interacted with Rv1768. Furthermore, Rv1768 and the PE domain of Rv1768 (Figures 4B,C), but not the PGRS domain of Rv1768 (Figure 4D), were found to be bound to S100A9 from macrophages via immunoblot analysis (Figures 4B–D).

Next, we analyzed the co-localization of Rv1768 and S100A9 by confocal microscopy analysis by co-transfecting pEGFP-C1-PE/pEGFP-C1-PGRS/pEGFP-C1-Rv1768 and pAsRed2-N1-S100A9 into RAW264.7 cells. After transfection for 24 h, we observed similar results: Rv1768 (green) and the PE domain (green) of Rv1768, but not the PGRS domain (green) of Rv1768, co-localized with S100A9 (red) in the cytoplasm of macrophages (Figure 4E and Supplementary Figure S2).

S100A9 and S100A8 usually act as dimers and danger-associated molecular pattern (DAMP) molecules (Muller et al., 2017); therefore, we examined whether Rv1768 could also interact with S100A8. We found that Rv1768 interacted with S100A8 in macrophages using Rv1768-His-Ni-NTA agarose pull-down, western blot (Figure 4F), and confocal microscopy analyses (Figure 4G). All the above data demonstrated that Rv1768 interacts with the S100A9/A8 heterodimer.

### Rv1768 Promotes Mycobacterial Survival via S100A8/A9

**FIGURE 5:**
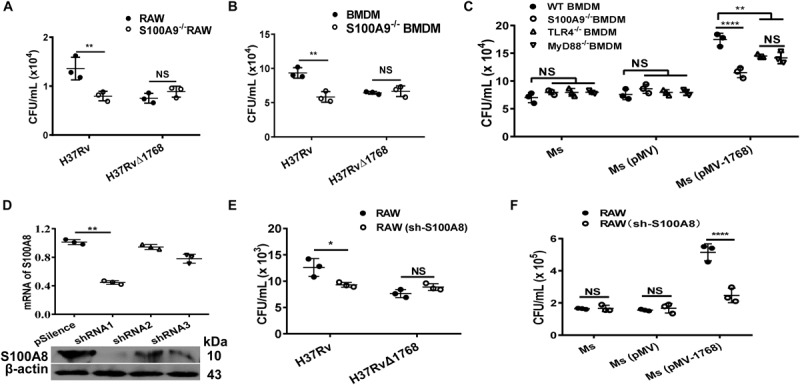
Rv1768 promotes mycobacteria survival in macrophage via S100A8 and S100A9. **(A,B)** Bacterial CFUs in WT RAW264.7 cells, S100A9^–/–^ RAW264.7 cells **(A)**, or WT BMDMs, S100A9^–/–^ BMDMs **(B)** infected with H37Rv or H37RvΔ1768 with mixed infection assay. **(C)** Bacterial CFUs of *Ms*, *Ms* (pMV) and *Ms* (pMV-1768) in the WT, S100A9^–/–^, TLR4^–/–^ or MyD88^–/–^ BMDMs with mixed infection assay. Two-way ANOVA with Tukey’s multiple comparison test was used to compare the means across multiple cell types and bacteria species. *p < 0.05, **p < 0.01, ****p < 0.0001. **(D)** RT-qPCR (up panel) and Western blot (lower panel) confirmation of the silencing efficiency of S100A8 shRNA. One-way ANOVA with Tukey’s multiple comparison test was used to compare the means across multiple groups. **(E,F)** CFU counting for mixed infection assays with RAW264.7 and S100A8-shRNA-transfected RAW264.7 cells infected with H37Rv, H37RvΔ1768 **(E)**, *Ms*, *Ms* (pMV) and *Ms* (pMV-1768) **(F).** Two-way ANOVA with Tukey’s multiple comparison test was used to compare the means across multiple cell types and bacteria species. The data are presented as mean ± SD (error bars). Data averaged from at least three independent experiments. *p < 0.05, **p < 0.01, ****p < 0.0001. NS, no significant difference.

RAW264.7 cells or BMDMs (WT or S100A9^–/–^) were infected with bacteria [H37Rv, H37RvΔ1768, *Ms*, *Ms* (pMV), or *Ms* (pMV-1768)] for the mixed infection assay. Results showed that more bacterial colonies were observed in H37Rv-infected WT RAW264.7 cells than in H37Rv-infected S100A9^–/–^ RAW264.7 cells (**p < 0.01, Figure 5A), but there was no significant difference in the bacterial loads of H37RvΔRv1768 (Figure 5A). Similar results were observed in BMDMs (for H37Rv, WT *vs.* S100A9^–/–^ RAW264.7 cells, **p < 0.01, Figure 5B). We also assessed the effects of *Ms* (pMV-1768) on WT BMDMs and S100A9/TLR4/MyD88 KO BMDMs. More bacterial colonies were observed in *Ms* (pMV-1768)-infected WT BMDMs than in S100A9^–/–^/TLR4^–/–^/MyD88^–/–^ BMDMs (**p < 0.01, Figure 5C), and fewer CFUs were observed in *Ms* (pMV-1768)-infected S100A9^–/–^ BMDMs than in TLR4^–/–^/MyD88^–/–^ BMDMs (**p < 0.01, Figure 5C). However, there were no significant differences in the bacterial loads of *Ms* or *Ms* (pMV) among these cells (Figure 5C). These results strongly suggest that Rv1768 promotes bacterial survival in macrophages via S100A9.

RAW264.7 cells were transfected with an effective short-hairpin RNA1 (shRNA1) against S100A8 and control shRNA2/3 (Figure 5D), then infected with bacteria for mixed infection assay. Results showed that more bacterial colonies were observed in the H37Rv and *Ms*(pMV-1768) groups compared to the H37RvΔ1768 and control [*Ms* and *Ms* (pMV)] groups in RAW264.7 cells (*p < 0.05, Figure 5E, ****p < 0.0001, Figure 5F), but there were no significant differences in CFUs in the shRNA1-S100A8-RAW264.7 cells (Figures 5E,F), demonstrating that Rv1768 also promotes bacterial survival in macrophages via S100A8.

These data strongly suggest that Rv1768 promotes mycobacterial invasion and survival in macrophages via its association with the S100A9/S100A8 heterodimer.

### Rv1768 Blocks the Interaction Between S100A9 and Toll-Like Receptor 4 (TLR4)

**FIGURE 6:**
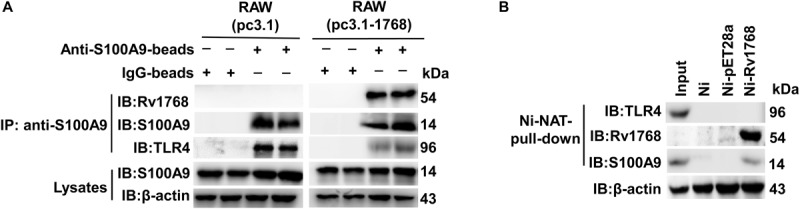
Rv1768 blocks S100A9 binding to TLR4 in macrophage. **(A)** Western blot analysis with anti-TLR4 and anti-S100A9 for anti-S100A9-beads-pull-down products of cell lysates from RAW264.7 cells transfected with pcDNA3.1-Rv1768 or pcDNA3.1. **(B)** Western blot analysis (with anti-TLR4, anti-Rv1768 and anti-S100A9) of Rv1768-His-Ni-NTA agarose pull-down products of RAW264.7 cell lysates.

Previous studies have shown that the S100A9/A8 heterodimer binds to TLR4 and acts as an endogenous activator to trigger subsequent signaling pathways, consequently causing an autocrine positive feedback loop and enhancing the LPS-induced up-regulation of inflammatory factors, such as chemokines and TNF-α (Vogl et al., 2007; Ehrchen et al., 2009; Nishikawa et al., 2017; Vogl et al., 2018). Therefore, we examined the relationship between Rv1768 and TLR4. Using IP and immunoblot analyses, we found that S100A9 was associated with TLR4, as expected (Figure 6A, left panel). Interestingly, S100A9 association with TLR4 decreased in the pcDNA-3.1-Rv1768-transfected RAW264.7 compared to the pcDNA3.1 empty vector-transfected RAW264.7 cells (Figure 6A, right panel). We further used the Rv1768-His-Ni-NTA agarose for pull-down and western blot analysis and found that Rv1768 could not directly bind to macrophage TLR4, while it could bind to S100A9 (Figure 6B). However, the control beads (Ni: empty Ni beads, Ni-pET28a: vector-conjugated Ni beads) could not pull-down S100A9 or TLR4 (Figure 6B). These data suggest that Rv1768 blocks the interaction between S100A9 and TLR4.

### Rv1768 Suppresses TLR4-MyD88-Nuclear Factor-κB (NF-κB)-TNF-α Signaling Axis of Macrophages via S100A9

**FIGURE 7:**
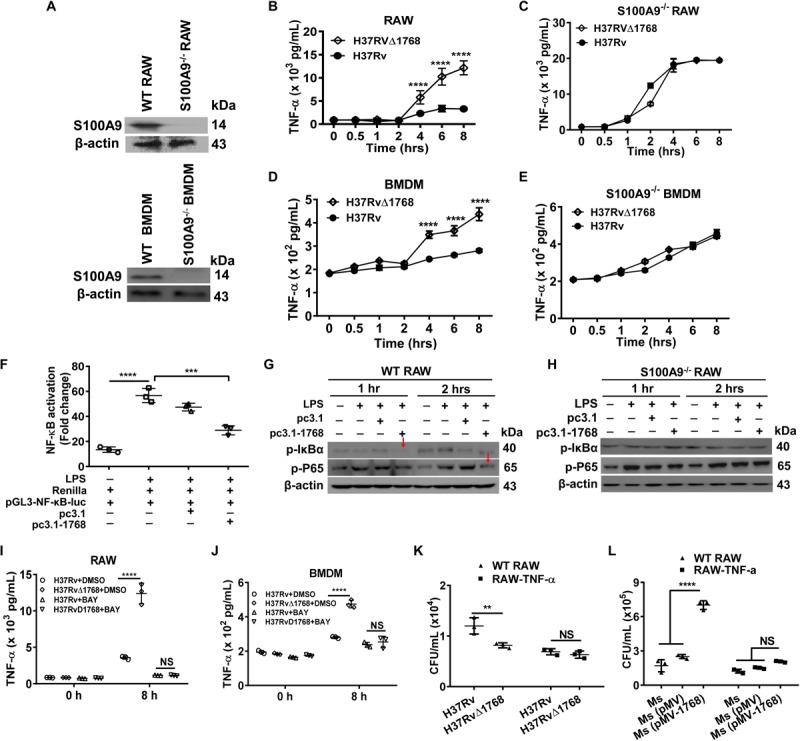
Rv1768 suppresses the NF-κB-TNF-α axis in macrophages. **(A)** Western blot analysis of S100A9 in WT RAW264.7 cells, BMDMs, S100A9^–/–^ RAW264.7 cells and S100A9^–/–^ BMDMs. **(B,C)** TNF-α in the supernatant of WT RAW264.7 cells **(B)** or S100A9^–/–^ RAW264.7 cells **(C)** infected with H37Rv or H37RvΔ1768 at different time. **(D,E)** TNF-α in the supernatant of WT BMDMs **(D)** or S100A9^–/–^ BMDMs **(E)** infected by H37Rv or H37RvΔ1768 at different time. Two-way repeated measures ANOVA with Tukey’s *post hoc* multiple comparison test was used to compare the means across multiple time points and multiple groups. **(F)** Dual luciferase assay analysis of the effect of Rv1768 on NF-κB activation in RAW264.7 cells. One-way ANOVA with Tukey’s multiple comparison test was used to compare the means across multiple groups. **(G,H)** Western blot analysis of the effect of Rv1768 on expression of p-p65 and p-IκBα after NF-κB activation by LPS in WT RAW264.7 **(G)** and S100A9^–/–^ RAW264.7 cells **(H)**. **(I,J)** TNF-α in the supernatant of RAW264.7 **(I)** or BMDMs **(J)** infected with H37Rv or H37RvΔ1768 with or without addition of NF-κB inhibitor BAY11-7082 (BAY, 10 μM, added 1 h before infection). Two-way ANOVA with Tukey’s multiple comparison test was used to compare the means across multiple bacteria species in the presence or absence of inhibitors at different time points. **(K,L)** Bacterial CFUs in RAW264.7 and RAW264.7-TNF-α cells infected with H37Rv or H37RvΔ1768 **(K)**, or infected with *Ms*, *Ms* (pMV) and *Ms* (pMV-1768) **(L)** with mixed infection assay. The data are presented as mean ± SD (error bars). Data averaged from at least three independent experiments. Two-way ANOVA with Tukey’s multiple comparison test was used to compare the means across multiple cell types and bacteria species. **p < 0.01, ***p < 0.001, ****p < 0.0001.

TNF-α is crucial for the control of TB. We assessed TNF-α levels in H37Rv- or H37RvΔ1768-infected WT or S100A9^–/–^ macrophages. S100A9^–/–^ RAW264.7 cells and S100A9^–/–^ C57BL/6 mice were constructed using the CRISPR/Cas9 system and confirmed by western blot analysis (Figure 7A). H37Rv infection induced much less secretion of TNF-α at 4 h post-infection compared to the H37RvΔ1768 group in WT RAW264.7 cells (****p < 0.0001, Figure 7B) and BMDMs (****p < 0.0001, Figure 7D). However, there was no difference in TNF-α secretion in both S100A9^–/–^ RAW264.7 cells (Figure 7C) and S100A9^–/–^ BMDMs (Figure 7E) between the H37Rv and H37RvΔ1768 groups, suggesting that Rv1768 suppresses TNF-α secretion by macrophages via S100A9.

NF-κB is a critical nuclear transcription factor that regulates inflammatory cytokine TNF-α expression. We performed dual luciferase assays to further determine the role of Rv1768 protein in macrophages during NF-κB activation. RAW264.7 cells were co-transfected with pGL3-NF-κB-luc (carrying the NF-κB promoter region), Renilla (used as an endogenous control), and pcDNA3.1-Rv1768 or pcDNA3.1. At 24 h post transfection, cells were treated with LPS to stimulate the activation of NF-κB. Compared to the empty vector (pcDNA3.1) transfection group, the transfection of pcDNA3.1-Rv1768 significantly suppressed NF-κB activation 1 h post-stimulation with LPS (***p < 0.001, Figure 7F). Western blot results also demonstrated that the transfection of Rv1768 significantly suppressed p-P65 and p-IκBα expression compared to the empty vector group in both RAW264.7 2 h post-infection (Figure 7G) but not in S100A9^–/–^ RAW264.7 cells (Figure 7H). However, we did not observe this inhibitory effect of Rv1768 on the activation of IFN-β transcription induced by poly I: C (data not shown). After the addition of an NF-κB inhibitor, BAY11-7082 (BAY), no significant difference in secreted TNF-α production was observed between H37Rv- and H37RvΔ1768-infected RAW264.7 cells (Figure 7I) and BMDMs (Figure 7J). However, there were no differences in the expression of pro-inflammatory cytokines IL-6 and IL-1β in the culture supernatants of WT and S100A9^–/–^ BMDMs between H37Rv and H37RvΔ1768 infection groups (Supplementary Figure S3). The above data suggest that Rv1768 suppresses the TLR4-MyD88-NF-κB-TNF-α axis of macrophages in a S100A9-dependent manner.

Next, both WT and RAW264.7 cells that stably express TNF-α (RAW264.7-TNF-α cells) were infected with H37Rv or H37RvΔ1768 using the mixed infection experiments as described above. Similarly, a greater number of bacterial CFUs were observed in the H37Rv group compared to the H37RvΔ1768 group in RAW264.7 cells (**p < 0.01, Figure 7K); however, there were no significant differences among these groups in RAW264.7 cells stably expressing TNF-α (RAW264.7-TNF-α cells) (Figure 7K). Similarly, the *Ms* (pMV-1768) group had a larger number of bacterial CFUs in RAW264.7 cells than the *Ms* and *Ms* (pMV) groups (****p < 0.0001, Figure 7L), but there were no differences in CFUs of RAW264.7-TNF-α cells among the *Ms*, *Ms* (pMV), and *Ms* (pMV-1768) groups (Figure 7L). These data further demonstrate that Rv1768 promotes bacterial survival by suppressing TNF-α production, at least in the early infection stages.

### Rv1768 Promotes Bacterial Survival via S100A9 *in vivo*

**FIGURE 8:**
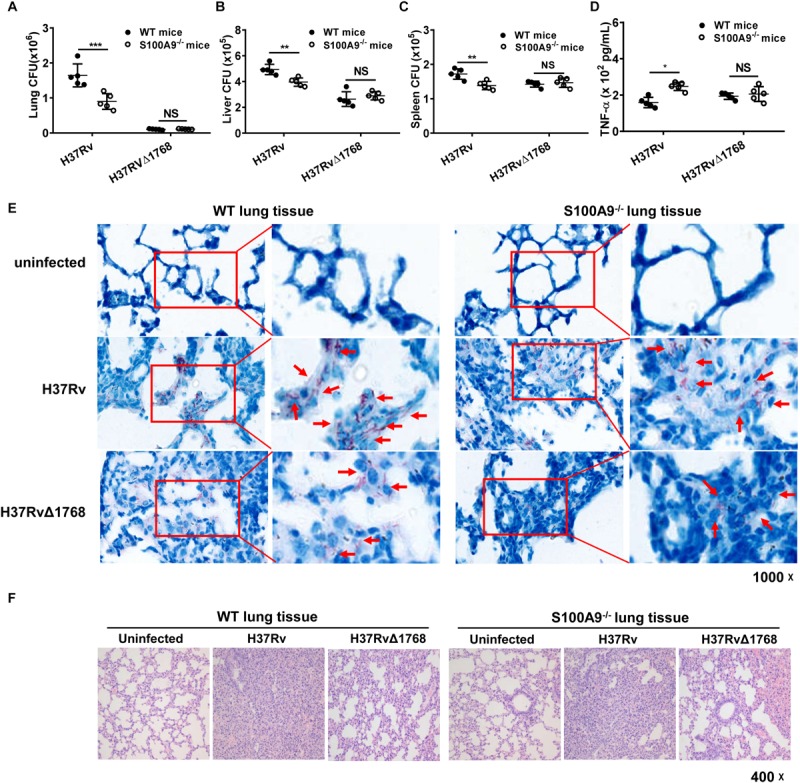
Rv1768 promotes mycobacterial survival *in vivo* via S100A9. **(A–C)** Bacterial burden in mouse lungs **(A)**, liver **(B)** and spleen **(C)** from WT or S100A9^–/–^ mice (*i.n*.) infected with H37Rv or H37RvΔ1768 strains (*n* = 5, each group) on Day 28 post infection. **(D)** Serum TNF-α in WT or S100A9^–/–^ C57BL/6 mice infected with H37Rv or H37RvΔ1768 on Day 28 post infection. The data are presented as mean ± SD (error bars). Data averaged from at least two independent experiments. Two-way ANOVA with Tukey’s multiple comparison test was used to compare the means across multiple cell/mouse types and bacteria species. *p < 0.05, **p < 0.01, ***p < 0.001, ****p < 0.0001. **(E,F)** Acid-fast staining **(E)** and H-E staining **(F)** of lung tissues from WT or S100A9^–/–^ mice infected with H37Rv, H37RvΔ1768 or uninfected controls.

Next, we examined whether Rv1768 promoted bacterial survival via S100A9 *in vivo*. Both WT and S100A9^–^/^–^ mice were *i.n.* infected with H37Rv or H37RvΔ1768 strains. On day 28 post-infection, bacterial loads in each organ were measured. Notably, H37Rv CFUs were significantly higher in the lungs (***p < 0.001, Figure 8A), liver (**p < 0.01, Figure 8B), and spleen (**p < 0.01, Figure 8C) of WT C57BL/6 mice than those in S100A9^–/–^ C57BL/6 mice, but there was no significant difference in the bacterial load of H37RvΔRv1768. These data suggest that Rv1768 promotes bacterial survival via S100A9 *in vivo* (Figures 8A–C). We also observed that the number of CFUs of WT H37Rv was higher than those of H37RvΔRv768 in S100A9^–/–^ C57BL/6 mice (Figures 8A–C), suggesting that other effectors of *M. tb* H37Rv may also promote the survival of macrophages in addition to Rv1768.

We further measured the effect of Rv1768 on pro-inflammatory cytokine expression. The serum TNF-α expression level was significantly lower in the H37Rv group compared to in the H37RvΔ1768 group in WT C57BL/6 mice (Figure 8D). However, there was no difference in serum TNF-α expression in S100A9^–/–^ mice between the H37Rv and H37RvΔ1768 groups (Figure 8D). Acid-fast staining analysis showed that more acid-fast-positive bacilli existed in the lung sections of H37Rv-infected mice compared to H37RvΔ1768-infected mice (Figure 8E). The abundance of acid-fast bacilli-positive H37Rv was much lower in the lung sections of S100A9^–/–^ mice compared to WT mice (Figure 8E). Histopathological analysis showed that the H37RvΔ1768 group had fewer pathological changes in lung tissues and fewer lesions with increased total cellular and neutrophilic infiltration compared to H37Rv in WT mice (Figure 8F). There were significant pathological changes between WT and S100A9^–/–^ mice infected with H37Rv, but no significant differences were observed between H37RvΔRv768-infected WT C57BL/6 mice and S100A9^–/–^ C57BL/6 mice (Figure 8F).

**FIGURE 9:**
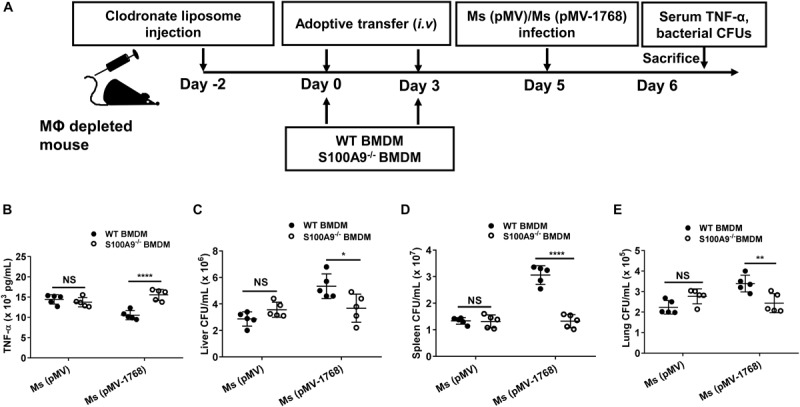
Adoptive transfer of S100A9^–/–^ macrophages attenuate the effect of TNF-a production and bacterial survival by Rv1768. **(A)** Procedure of the WT and S100A9^–/–^ BMDM adoptive transfer experiment. **(B)** Serum TNF-α in *Ms* (pMV)-infected or *Ms* (pMV-1768)-infected mice adoptively transferred with WT or S100A9^–/–^ BMDMs. **(C–E)** Bacterial burden in the liver **(C)**, spleen **(D)** and lungs **(E)** from *Ms* (pMV)-infected or *Ms* (pMV-1768)-infected mice adoptively transferred with WT or S100A9^–/–^ BMDMs (*n* = 5, each group). The data are presented as mean ± SD (error bars). Data averaged from at least two independent experiments. Two-way ANOVA with Tukey’s multiple comparison test was used to compare the means across multiple cell types and bacteria species. *p < 0.05, **p < 0.01, ****p < 0.0001.

We further adoptively transferred WT or S100A9^–/–^ mouse-derived BMDMs into macrophage-depleted WT C57BL/6 mice (Figure 9A and Supplementary Figure S4). Then, the mice were *i.v.* infected with *Ms* (pMV) and *Ms* (pMV-Rv1768). *Ms* is a fast-growing bacterium. On day 1 post-infection, the mice were euthanized, and serum TNF-α production and bacterial burden in different organs were measured. Our results showed that *Ms* (pMV-1768) infection inhibited serum TNF-α production (****p < 0.01, Figure 9B) and promoted bacterial survival compared to the *Ms* (pMV) control group, as illustrated by the increased CFUs in different organs, including the liver (*p < 0.001, Figure 9C), spleen (****p < 0.0001, Figure 9D), and lungs in the WT BMDM adoptive transfer mice (**p < 0.01, Figure 9E). However, no significant differences in serum TNF-α production and liver, spleen, and lung CFUs were observed among the groups in the S100A9^–/–^ BMDM adoptive transfer mice (Figures 9B–E). Notably, bacterial burdens in the *Ms* (pMV-1768)-infected WT BMDM adoptive-transfer mice were much higher in the liver (*p < 0.05, Figure 9C), spleen (****p < 0.0001, Figure 9D), and lungs (**p < 0.01, Figure 9E) than in S100A9^–^/^–^ BMDM adoptive-transfer mice. However, there were no differences in bacterial CFUs in the *Ms* (pMV) group, suggesting that Rv1768 promotes bacterial survival via S100A9. These data strongly suggest that Rv1768 can promote mycobacterial growth and suppress TNF-α production *in vivo* via S100A9 of macrophages.

### Rv1768 Disturbs the Metabolism of Arachidonic acid via S100A9

**FIGURE 10:**
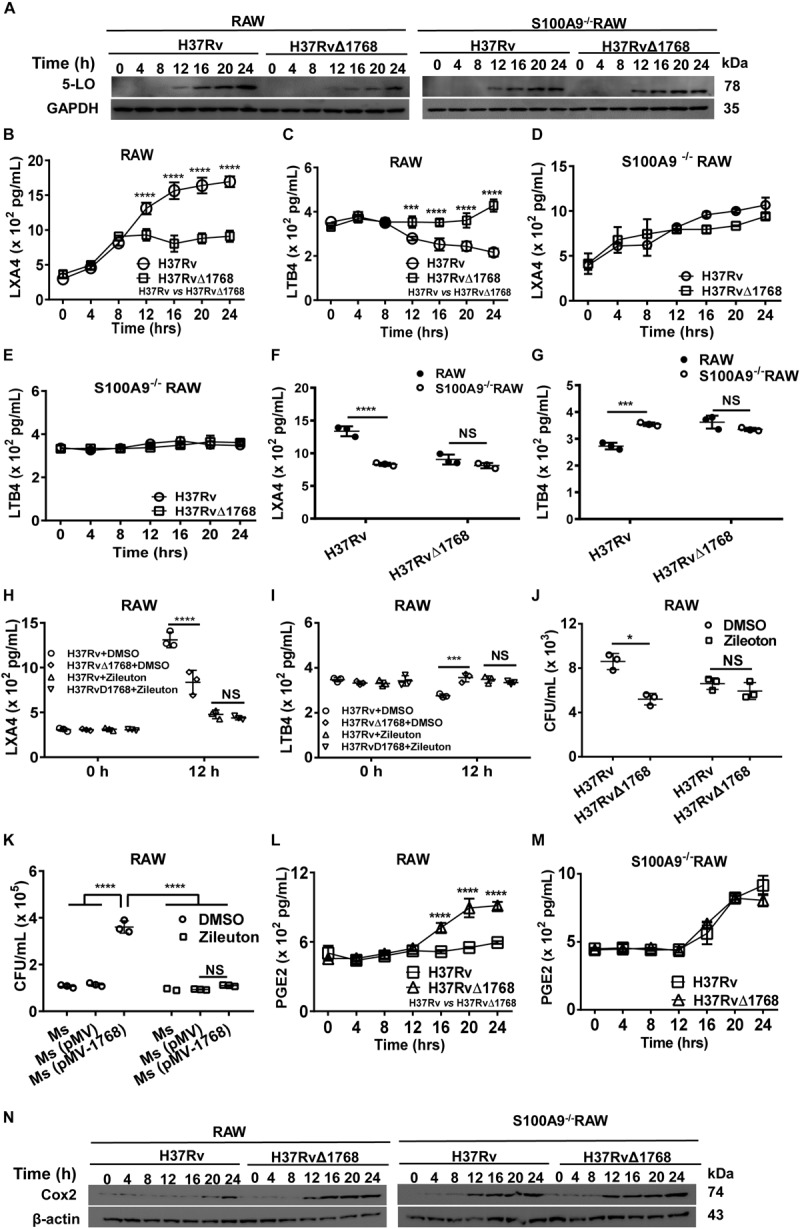
Rv1768 disturbs the metabolism of arachidonic acid via S100A9. **(A)** Western blot analysis of 5-LO in WT and S100A9^–/–^ RAW264.7 cells at different time points post infection with H37Rv or H37RvΔ1768. **(B–E)** ELISA assessment of LXA4 **(B,D)** and LTB4 **(C,E)** levels in the supernatants from WT RAW264.7 cells **(B,C)** and S100A9^–/–^ RAW264.7 **(D,E)** cells infected with H37Rv or H37RvΔ1768. Two-way repeated measures ANOVA with Tukey’s *post hoc* multiple comparison test was used to compare the means across multiple time points and multiple groups. **(F,G)** The LXA4 **(F)** and LTB4 **(G)** in the supernatants from WT and S100A9^–/–^ RAW264.7 cells infected with H37Rv or H37RvΔ1768 for 12 h **(H,I)** LXA4 **(H)** and LTB4 **(I)** in the supernatants of WT RAW264.7 cells infected with H37Rv or H37RvΔ1768 with or without the presence of 5-LO inhibitor Zileuton (0.5 μM, added 0.5 h before infection). Two-way analysis of variance (ANOVA) with Tukey’s *post hoc* multiple comparison test was used to compare the means across multiple groups. **(J)** CFUs of H37Rv and H37RvΔ1768 in RAW264.7 cells at 12 h with or without Zileuton. **(K)** CFUs of *Ms*, *Ms* (pMV) and *Ms* (pMV-1768) in RAW264.7 cells at 12 h with or without Zileuton. Two-way ANOVA with Tukey’s multiple comparison test was used to compare the means across bacteria species in the presence or absence of inhibitors. **(L,M)** PGE2 in the supernatant of WT **(L)** and S100A9^–/–^ RAW264.7 **(M)** cells infected with H37Rv or H37RvΔ1768 at different time. Two-way repeated measures ANOVA with Tukey’s *post hoc* multiple comparison test was used to compare the means across multiple time points and multiple groups. **(N)** Western blot of Cox2 in WT and S100A9^–/–^ RAW264.7 cells infected with H37Rv or H37RvΔ1768 at different time. The data are presented as mean ± SD (error bars). Data averaged from at least three independent experiments. *p < 0.05, **p < 0.01, ***p < 0.001, ****p < 0.0001.

Recent findings indicate that 5-lipoxygenase (5-LO), an enzyme required for the production of the lipid mediator leukotrienes (LTs) and lipoxins, negatively regulates the Th1 response during intracellular bacterial infection (Bafica et al., 2005; Mishra et al., 2018). Our results showed that 5-LO expression increased 12 h post-infection with H37Rv in RAW264.7 cells compared to the H37RvΔ1768 infection group, as determined by western blot analysis (Figure 10A, left). In contrast, 5-LO expression showed no significant changes between H37Rv- and H37RvΔ1768-infection in S100A9^–/–^ RAW264.7 cells (Figure 10A, right), suggesting that Rv1768 stimulates macrophage 5-LO expression after infection for 12 h, and that this stimulation depends on S100A9.

LTs produced from arachidonic acid by the action of 5-LO are classical mediators of inflammatory responses (Herb et al., 2008). We further determined the effect of Rv1768 on the expression of LXA4 and LTB4 in macrophages. The levels of LXA4 and LTB4 in the culture supernatants of RAW264.7 cells were measured by ELISA. Compared to the H37RvΔ1768 infection group, H37Rv infection promoted the production of LXA4 (****p < 0.0001, Figure 10B) and inhibited LTB4 (***p < 0.001, Figure 10C) in WT RAW264.7 cells after 12 h of infection. However, no significant difference in LXA4 and LTB4 production was observed in S100A9^–/–^ RAW264.7 cells between the H37Rv and H37RvΔ1768 infection groups (Figures 10D,E). H37Rv infection induced much higher LXA4 (****p < 0.0001, Figure 10F) and lower LTB4 production in WT RAW264.7 cells (***p < 0.001, Figure 10G) after infection for 12 h than in the S100A9^–/–^ RAW264.7 cells. However, there was no significant difference in LXA4 and LTB4 production in H37RvΔ1768 infection between WT and S100A9^–/–^ RAW264.7 cells. These data suggest that Rv1768 enhances the expression of the anti-inflammatory lipid mediator LXA4 and inhibits the expression of the pro-inflammatory lipid mediator LTB4 in macrophages via S100A9.

We further used a selective 5-LO inhibitor, Zileuton, to examine whether the changes in LXA4 and LTB4 were induced by 5-LO expression after Rv1768 stimulation. In the presence of Zileuton, the differences in LXA4 and LTB4 expression between H37Rv and H37RvΔ1768 infection were alleviated after infection for 12 h (Figures 10H,I), suggesting that Rv1768 stimulates 5-LO expression, thus, affecting LXA4 and LTB4 expression levels. We also examined whether Rv1768 promoted bacterial survival by stimulating 5-LO expression. At 12 h post infection, we found that H37Rv infection caused much higher bacterial counts in RAW264.7 cells compared to the H37RvΔ1768 group, but not in the presence of Zileuton (Figure 10J). Similarly, compared with control groups (*Ms* and *Ms*[pMV]), *Ms* (pMV-1768) infection resulted in much higher bacterial counts in RAW264.7 cells, but not in the presence of Zileuton (Figure 10K). These results suggest that Rv1768 promotes the mycobacterial survival of macrophages by enhancing 5-LO expression, at least at the late infection stage (after infection for 12 h).

We also determined the effects of Rv1768 on Cox-2 expression and PGE2 synthesis. Both WT and S100A9^–/–^ RAW264.7 cells were infected with H37Rv or H37RvΔ1768. We then detected Cox-2 and secreted PGE2 expression by western blotting and ELISA, respectively, at different time points (0, 2, 4, 6, 8, 12, 16, 20, and 24 h post infection). Our results showed that H37Rv infection inhibited both PGE2 production (****p < 0.0001, Figure 10L) and Cox-2 expression (Figure 10N, left panel) in WT RAW264.7 cells 12 h post-infection, compared to those in S100A9^–/–^ RAW264.7 cells (Figures 10L,N). However, there were no significant differences in either PGE2 production (Figure 10M) or Cox-2 expression (Figure 10N, right panel) between the H37Rv and H37RvΔ1768 groups in S100A9^–/–^ RAW264.7 cells. These results suggested that Rv1768 inhibited Cox-2 expression via S100A9, thus, decreasing PGE2 expression, at least at the later stage of infection (12 h post-infection).

## Discussion

*Mycobacterium tuberculosis*, as an intracellular pathogen, can invade and survive in macrophages. It can persist in host macrophages for long periods of time. Several studies have shown that mycobacteria establishes a niche by evading immune recognition via multiple mechanisms, including masking, establishing dormancy by manipulating immune responses, altering innate immune cell fate, enhancing granuloma formation, and developing antibiotic tolerance (Peddireddy et al., 2017). In this study, we identified a novel RD14-encoded protein, Rv1768, that significantly promoted the invasion and survival of *M. tb* in both macrophages and mice. Our findings may enhance our understanding of the pathogenesis mechanism of virulent *M. tb*.

*Mycobacterium tuberculosis* PE_PGRSs represent a family of complex and interesting proteins whose roles and functions remain elusive. Rv1768 belongs to the PE_PGRS subfamily, PE_PGRS31 (Meena, 2015). PE_PGRSs are surface-exposed proteins restricted to the *M. tb* complex and a few other pathogenic mycobacteria that have been implicated in interactions with host components (Espitia et al., 1999; Brennan et al., 2001; Brennan and Delogu, 2002). However, there have been few studies regarding Rv1768 (PE_PGRS31). Our present results show that Rv1768 is located in the bacterial CW and only exits in H37Rv and H37Ra, not in *M. avium*, *Ms, M. marinum*, *M. intracellulare*, or BCG. Some other members of the *M. tb* PE_PGRS family, for example, PE_PGRS33, are surface-exposed proteins that interact with TLR2 on host macrophages to induce inflammatory signals and promote entry into macrophages (Minerva et al., 2017). PE_PGRS3 mediates adhesion and persistence in host cells (De Maio et al., 2018). The expression of the recombinant protein in *Ms*, which does not possess any PE_PGRS proteins or an ESX-5 secretion system, has been widely used to investigate these proteins, and the results obtained have provided some relevant information (Abdallah et al., 2008). *Ms* is often used as an alternative for *M. tb* in experimental TB, because *Ms* offers some technical benefits, such as a shorter generation time and negligible risk to laboratory workers compared to *M. tb*. Several phenotypes, including total cytoplasmic ribosome number, antigenicity, acid-fastness, and the mechanism of drug resistance in *Ms* are also different to those in *M. tb* (Yamada et al., 2018). Approximately 30% of *M. tb* proteins lack conserved orthologs in *Ms* compared to 3% being absent in BCG (Altaf et al., 2010). Therefore, *Ms* does not completely replace *M. tb* for some function studies. In the present study, we used recombinant *Ms* (pMV261-Rv1768) and controls (*Ms* and *Ms* pMV261) for preliminary verification, and further used *M. tb* H37Rv and *M. tb* H37RvΔ1768 to confirm our hypothesis.

Previous studies involving PE_PGRS proteins have focused on their ability to facilitate the uptake of mycobacteria by macrophages (Yang et al., 2017). However, little is known regarding their function after the pathogen has entered the host macrophages. We are interested in the intracellular function of Rv1768 in macrophages. Our present results showed that *Ms* carrying Rv1768 promoted bacterial survival *in vivo* and *in vitro*. The Rv1768-deficient strain (H37RvΔ1768) displayed much lower bacterial CFUs in macrophages and in the lungs, spleen, and liver compared to H37Rv in murine infection models, suggesting that *M. tb* Rv1768 promoted mycobacterial survival in both macrophages and mice.

We found that Rv1768 inhibited the TLR4-MyD-88-NF-κB-TNF-α signaling axis via S100A9. TLR4 triggers the activation of NF-κB via the MyD88-dependent pathway, resulting in the up-regulation of proinflammatory mediators. The NF-κB transcription factor is essential for inducing the expression of a variety of inflammatory genes in response to a range of pathogens and inflammatory cytokines. TNF-α is regulated by the NF-κB signaling pathway in the process of restricting bacterial survival in granulomas and aggregating bacteria and immune cells within the lungs (Fallahi-Sichani et al., 2012). Our study showed that Rv1768 specifically inhibits the TLR4-MyD-88-NF-κB-TNF-α signaling axis via S100A9 and inhibits the expression of the TNF-α pro-inflammatory cytokine, but has no effect on the expression of the pro-inflammatory cytokines IL-6 and IL-1β. TNF-α plays a crucial role in the control of TB (Lin et al., 2007; Di Paolo et al., 2015). TNF-α activates macrophages, recruits them to the site of infection, and participates in granuloma formation (Lin et al., 2007). Therefore, Rv1768 promotes mycobacterial survival via S100A9, primarily by inhibiting the TLR4-MyD88-NF-κB-TNF-α innate immune signaling axis.

S100A9 is a member of the S100 family of proteins that is expressed in a wide range of cells, and is especially abundant in neutrophils, monocytes, and macrophages (Wang et al., 2018). S100A9 is also referred to as myeloid-related protein 14. Normally, S100A9 forms a heterodimer with S100A8, and the heterodimer plays multiple roles in the cell by facilitating leukocyte arachidonic acid trafficking and metabolism (Averill et al., 2011), modulating the tubulin-dependent cytoskeleton during the migration of phagocytes (Alexaki et al., 2017), and activating neutrophil NADPH-oxidase (Benedyk et al., 2007). S100A9 is also a member of the DAMP protein family and is released by activated phagocytic cells, such as neutrophils, macrophages, and endothelial cells. The S100A8/S100A9 heterodimer induces the translocation of the MyD88 adaptor protein from the cytoplasmic space to the TLR4 receptor complex, thus, activating extracellular signal-regulated kinases, c-Jun N-terminal kinases, p38, and NF-κB (Xia et al., 2012). The activation of NF-κB can induce pro-inflammatory signal transduction and, thus, lead to the activation of the innate immune system. Our findings show that Rv1768 binding to S100A9 blocks S100A9 association with TLR4. Deficiency of S100A9 or S100A8 dramatically decreases the effect of Rv1768 on bacterial survival in macrophages and mice. These data suggest that the Rv1768-S100A9 interaction might predominately suppress TLR4-MyD88-NF-κB-TNF-α signaling and promote mycobacterial survival of macrophages at early *in vitro* and *in vivo* stages.

Our study also showed that the Rv1768-S100A9 interaction disturbed the metabolism of arachidonic acid and promoted mycobacterial survival. 5-LO is the key enzyme in the biosynthesis of LTs and LXs from arachidonic acid. The balance between the lipid mediators LXA4 and LTB4 represents at least one of the factors that dictate susceptibility or protection. The disturbance of the 5-LO-dependent fine equilibrium of LTs and LXs could compromise the homeostasis of LXA4 and LTB4, leading to *M. tb* survival and dissemination (Herb et al., 2008; Tobin et al., 2012; Dietzold et al., 2015; Pedruzzi et al., 2016; Mishra et al., 2018). Consistently, our study has shown that Rv1768 induces 5-LO expression, consequently promoting LXA4 expression and inhibiting LTB4 expression. The inhibition of 5-LO by Zileuton alleviated the promotion of bacterial survival by Rv1768. LXA4 induced by Rv1768 further inhibited Cox-2 expression, thus, decreasing PGE2 expression via S100A9 after mycobacterial infection for 12 h (Figure 9). LXA4 suppresses Cox-2 expression and decreases PGE2 synthesis, thereby, deviating the infected macrophages toward necrosis and promoting the dissemination of mycobacteria (Chen et al., 2008; Kumar et al., 2014). Therefore, Rv1768 up-regulates 5-LO, leading to a disturbance in the balance between LXA4 and LTB4, and consequently promoting mycobacterial growth and dissemination. This effect depends on S100A9, at least in the later stages of infection (Figure 10).

The function of Rv1768 (PE_PGRS31) of H37Rv has not previously been reported. Our present study is the first to reveal that *M. tb* Rv1768 promotes mycobacterial survival in macrophages by regulating NF-κB-TNF-α signaling and arachidonic acid metabolism via S100A9. We found that Rv1768 and the PE domain of Rv1768 (Figures 4B,C), but not the PGRS domain of Rv1768 (Figure 4D), bound to S100A9. However, the specific domain of S100A9 that interacts with Rv1768 and the mechanism underlying the interaction between Rv1768 and S100A8/A9 still needs to be further investigated.

**FIGURE 11:**
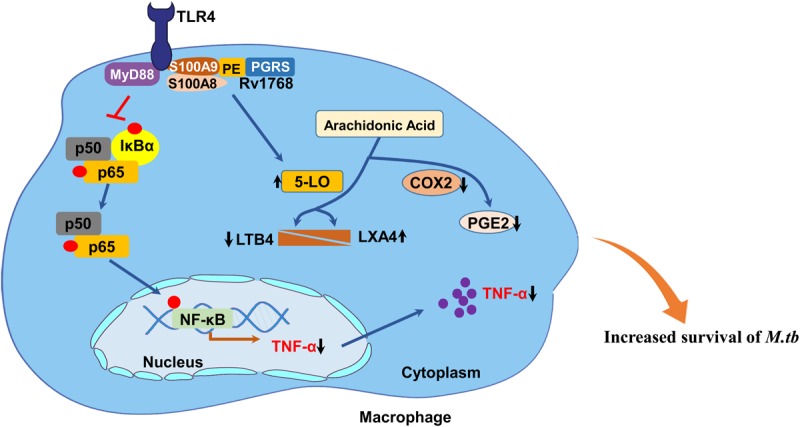
Proposed model of the mechanism of Rv1768/PE_PGRS31 in promotion of mycobacterial survival in macrophage. *M. tb* Rv1768 promotes mycobacterial survival in macrophage through the regulation of NF-κB-TNF-α signaling and metabolism of arachidonic acid via S100A9.

In summary, this study clarifies the role of Rv1768 (PE_PGRS31) in enhancing bacterial survival and immune escape by regulating TLR4-MyD88-NF-κB-TNF-α signaling and arachidonic acid metabolism (Figure 11). This study suggests that Rv1768 interacts with S100A9/A8 to suppress both the NF-κB-TNF-α signaling pathway during the early infection stage (about 4 h) and the metabolism of arachidonic acid at the late infection stage (after 12 h) to promote the intracellular survival of mycobacteria, and that disturbing the interaction between Rv1768 and S100A9/S100A8 may be a potential therapeutic target in TB.

## Data Availability Statement

The original contributions presented in the study are included in the article/Supplementary Material, further inquiries can be directed to the corresponding author.

## Ethics Statement

The animal study was reviewed and approved by Chinese National Laboratory Animal-Guideline for Ethical Review of Animal Welfare and approved by the Institutional Animal Care and Use Committee (IACUC) of Wuhan University (NO. 18021B) and the Second Military Medical University of Shanghai (No. 18002).

## Author Contributions

SL, YX, WL, and YD performed the experiments and analyzed the data. SL and YD performed all infection experiments. SL and ZX assessed colocalization in the cell lines. HX contributed H-E stain expertise. X-LZ initiated the study, analyzed the data, and created and revised the manuscript. All authors contributed to and approved the final version of the manuscript.

## Conflict of Interest

The authors declare that the research was conducted in the absence of any commercial or financial relationships that could be construed as a potential conflict of interest.

## Supplementary Material

FIGURE S1 Construction of H37RvΔ1768 by phage-mediated allelic exchange. **(A)** Schematic showing the construction of H37RvΔ1768 strain. LFP, left forward primer; LRP, left reverse primer; RFP, right forward primer; RRP, right reverse primer; LVFP, left verification forward primer; LVRP, left verification reverse primer; RVFP, right verification forward primer; RVRP: right verification reverse primer; Res, resolvase specific site for antibiotic resistance cassette removal. **(B)** Agarose electrophoresis of the homologous left and right arm. **(C)** PCR amplification products from H37Rv and H37RvΔ1768. **(D)** Representative colonial morphology of H37Rv and H37RvΔ1768. **(E)** Representative flow cytometry analysis of the purity of isolated mouse BMDMs. **(F)** Agarose electrophoresis of Rv1768 PCR products in recombinant *M. smegmatis*. **(G)** Representative colonial morphology of *Ms*, *Ms* (pMV) and *Ms* (pMV-1768).

FIGURE S2 Confocal microscope analysis of the co-localization between Rv1768/1768-PE/1768-PGRS and S100A9/S100A8 in RAW264.7 cell. **(A–C)** Confocal microscopy analysis of co-localization between Rv1768 and S100A9 in RAW264.7 cells. **(A)** RAW264.7 were co-transfected with pAsRed2-N1-S100A9 and pEGFP-C1-PE, **(B)** RAW264.7 were co-transfected with pAsRed2-N1-S100A9 and pEGFP-C1-PGRS, **(C)** RAW264.7 were co-transfected with pAsRed2-N1-S100A9 and pEGFP-C1-Rv1768. At 24 h post transfection, the cellular nuclei were labeled by DAPI. Confocal images were taken with a Leica-LCS-SP8-STED confocal system. **(D)** Confocal microscopy analysis of the co-localization between Rv1768 and S100A8 in RAW264.7 cells. The cells were co-transfected with pEGFP-Rv1768 and pAsRed2-N1-S100A8.

FIGURE S3 IL-6 and IL-1β in the supernatant of macrophages infected with H37Rv or H37RvΔ1768 at different time. **(A,B)** IL-6 levels in the supernatants of WT **(A)** and S100A9^–/–^ BMDMs **(B)** infected with H37Rv or H37RvΔ1768 at MOI = 10 for different time. **(C,D)** IL-1β levels in the supernatants of WT **(C)** and S100A9^–/–^ BMDM **(D)** infected with H37Rv or H37RvΔ1768 at MOI = 10 for different time. Two-way repeated measures ANOVA with Tukey’s *post hoc* multiple comparison test was used to compare the means across multiple time points and multiple groups. The data are presented as mean ± SD (error bars). Data averaged from at least three independent experiments. **p* < 0.05, ***p* < 0.01, ****p* < 0.001, *****p* < 0.0001.

FIGURE S4 FCM analysis for the murine macrophage deal with clodronate liposome **(A)** Negative control. **(B)** BMDM from control liposome injected mouse. **(C)** BMDM from clodronate liposome injected mouse.
