# Supplementary material for: PE_PGRS31-S100A9 Interaction Promotes Mycobacterial Survival in Macrophages Through the Regulation of NF-κB-TNF-α Signaling and Arachidonic Acid Metabolism
Source: Front Microbiol. 2020 May 8;11:845. doi: 10.3389/fmicb.2020.00845 (PMC7225313; doi:10.3389/fmicb.2020.00845)
Supplement: Supplementary file 1 [file Table_1.docx]

**Supplementary Table 1. Premiers used in this study**

| **Name** | **Sequence** | **Restriction Endonuclease** |
| --- | --- | --- |
| pET28a-Rv1768 | Forward: 5'-AGGGAATTCATGTCCTATCTCGTCGTGG-3' | *Eco*RI |
|  | Reverse: 5'-ATTAAGCTTCCCGGGCATCCCATT-3' | *Hin*dIII |
|  |  |  |
| pET28a-PE-1768 | Forward: 5'-AGGGAATTCATGTCCTATCTCGTCGTGG-3' | *Eco*RI |
|  | Reverse: 5'-ATTAAGCTTCCCGCCGGACCCGCCGTT-3' | *Hin*dIII |
|  |  |  |
| pET28a-PGRS-1768 | Forward: 5'-AGGGAATTCATGGTCAACCAGGCCGGT-3' | *Eco*RI |
|  | Reverse: 5'- ATTAAGCTTCCCGGGCATCCCATT-3' | *Hin*dIII |
|  |  |  |
| pAsRed2-N1-S100a9 | Forward: 5'-TTAAAGCTTGCATGGCCAACAAAGCACCTTCTC-3' | *Hin*dIII |
|  | Reverse: 5'-TTAGGATCCCTTCCCACAGCCTTTGCCATGA-3' | *Bam*HI |
|  |  |  |
| pEGFP-C1-RV1768 | Forward: 5'-AGGGAATTCGATGTCCTATCTCGTCGTGG-3' | *Eco*RI |
|  | Reverse: 5'- ATTTCTAGACCCGGGCATCCCATT-3' | *Xba*I |
|  |  |  |
| pEGFP-C1-PE-Rv1768 | Forward: 5'-AGGGAATTCGATGTCCTATCTCGTCGTGG-3' | *Eco*RI |
|  | Reverse: 5'-ATTTCTAGACCCGCCGGACCCGCCGTT-3' | *Xba*I |
|  |  |  |
| pEGFP-C1-PGRS-Rv1768 | Forward: 5'-AGGAAGCTTCGATGGTCAACCAGGCCGGT-3' | *Hin*dIII |
|  | Reverse: 5'-ATTGAATTCCCCGGGCATCCCATT-3' | *Eco*RI |
|  |  |  |
| pMV261-Rv1768 | Forward: 5'-AGGGAATTCATGTCCTATCTCGTCGTGG-3' | *Eco*RI |
|  | Reverse: 5'- ATTAAGCTTCCCGGGCATCCCATT-3' | *Hin*dIII |
|  |  |  |
| pcDNA3.1-Rv1768 | Forward: 5'-AGGGAATTCGCCATGGCCTCCTATCTCGTCGTGG-3' | *Eco*RI |
|  | Reverse: 5'-ATTAAGCTTCCCGGGCATCCCATT-3' | *Hin*dIII |
|  |  |  |
|  |  |  |
| Guide RNA1 for S100A9 KO RAW  RAW264.7 cells | 5'-CACCGTTGATGGAAGGTGTCGATGA-3' |  |
| Guide RNA2 for S100A9 KO RAW | 5'-CAACTACCTTCCACAGCTACTCAAA-3' |  |
|  |  |  |
| Guide RNA 1 for S100A9 KO mice | 5'-ATGCTGCGCTCCATCTGAGAAGG-3' |  |
| Guide RNA 2 for S100A9 KO mice | 5'-CAAAGCTCAGCTGATTGTCCTGG-3' |  |
|  |  |  |
| S100A9 KO mice PCR-confirmation F1 | Forward: 5'-GTATATGTGGAGGGAAGCTGTCTC-3' |  |
| S100A9 KO mice PCR-confirmation R1 | Reverse: 5'-GTGAAAGGAGGCAGAAAGGACATG-3' |  |
| S100A9 KO mice PCR-confirmation R2 | Reverse: 5'-CTATCCACAGAACTGTCTCACCATC-3' |  |
|  |  |  |
| S100A9 KO mice sequence primer | 5'-GTGAAAGGAGGCAGAAAGGACATG-3' |  |
|  |  |  |
| pSilencer U6-sh-S100A8 1 | Oligo 1: 5'-GCTCCGTCTTCAAGACATCGTT TCA-3' |  |
|  | Oligo 2: 5'-AGCTTACGATGTCTTGAAGACGGAGCTTTTTT-3' |  |
|  | Oligo 3:5'-AATTAAAAAAGCTCCGTCTTCAAGACATCGTA-3' |  |
|  | Oligo 4:5'-AGCTTGAAACGATGTCTTGAAGACGGAGCGGCC-3' |  |
|  |  |  |
| pSilencer U6-sh-S100A8 2 | Oligo 1: 5'-GACATCGTTTGAAAGGAAATCTTCA-3' |  |
|  | Oligo 2: 5'-GATTTCCTTTCAAACGATGTCTTTTTT-3' |  |
|  | Oligo 3: 5'-AATTAAAAAAGACATCGTTTGAAAGGAAATCA-3' |  |
|  | Oligo 4: 5'-AGCTTGAAGATTTCCTTTCAAACGATGTCGGCC-3' |  |
|  |  |  |
| pSilencer U6-sh-S100A8 3 | Oligo 1: 5'-GGAAATCTTTCGTGACAATGCTTCA-3' |  |
|  | Oligo 2: 5'-AGCTT GCATTGTCACGAAAGATTTCCTTTTTT-3' |  |
|  | Oligo 3: 5'-AATTAAAAAAGGAAATCTTTCGTGACAATGCA-3' |  |
|  | Oligo 4: 5'-AGCTTGAAGCATTGTCACGAAAGATTTCCGGCC-3' |  |
|  |  |  |
| Left homologous arm | Forward:5'-TTTTTTTTCCATAGATTGGGCGGGTTGCTATTCGGCCAA -3' | *Van*91I |
|  | Reverse: 5'- TTTTTTTTCCATCTTTTGGGGATGATCGCGTTGTGGACC -3' | *Van*91I |
|  |  |  |
| Right homologous arm | Forward: 5'- TTTTTTTTCCATAGATTGGGCGGGTTGCTATTCGGCCAA -3' | *Van*91I |
|  | Reverse: 5'- TTTTTTTTCCATCTTTTGGGGATGATCGCGTTGTGGACC -3 | *Van*91I |
|  |  |  |
| Left verification primer LVFP | Forward: 5'-CCCGAAGCCATCAATAACC-3' |  |
|  | Reverse: 5'-GTGGACCTCGACGACCCTAG-3' |  |
|  |  |  |
| Right verification primer RVFP | Forward: 5'-TGGATCTCTCCGGCTTCACC-3' |  |
|  | Reverse: 5'-GGTAATCAACGGCTCCTGC-3' |  |
|  |  |  |
| pAsRed2-N1-S100a8 | Forward: 5'-TTAAAGCTT CATGCCGTCTGAACTGGAGAAGGCCT-3’ | *Hin*dIII |
|  | Reverse: 5'-TTAGGATCC CTACTCCTTGTGGCTGTCTTTGTG-3’ | *Bam*HI |
