# Supplementary material for: PE_PGRS31-S100A9 Interaction Promotes Mycobacterial Survival in Macrophages Through the Regulation of NF-κB-TNF-α Signaling and Arachidonic Acid Metabolism
Source: Front Microbiol. 2020 May 8;11:845. doi: 10.3389/fmicb.2020.00845 (PMC7225313; doi:10.3389/fmicb.2020.00845)
Supplement: Supplementary file 2 [file Table_2.docx]

**Supplementary Table 2. Mass spectrometry analysis of potential proteins binding with Rv1768 in RAW264.7 cells.**

| **No.** | **Protein name** | **Protein ID (UniProt)** | **Numbers of unique peptides** | **species** | **Protein Score** |
| --- | --- | --- | --- | --- | --- |
| 1 | PKM | P52480 | 21 | MOUSE | 86.95 |
| 2 | S100A9 | Q545V2 | 25 | MOUSE | 76.76 |
| 3 | TOP1 | Q04750 | 18 | MOUSE | 68.02 |
| 4 | DHX15 | Q3UKJ6 | 18 | MOUSE | 59.40 |
| 5 | HNRNPL | G5E924 | 25 | MOUSE | 57.28 |
| 6 | LDHA | Q564E2 | 18 | MOUSE | 48.84 |
