# Supplementary figures and images for: PE_PGRS31-S100A9 Interaction Promotes Mycobacterial Survival in Macrophages Through the Regulation of NF-κB-TNF-α Signaling and Arachidonic Acid Metabolism
Source: Front Microbiol. 2020 May 8;11:845. doi: 10.3389/fmicb.2020.00845 (PMC7225313; doi:10.3389/fmicb.2020.00845)

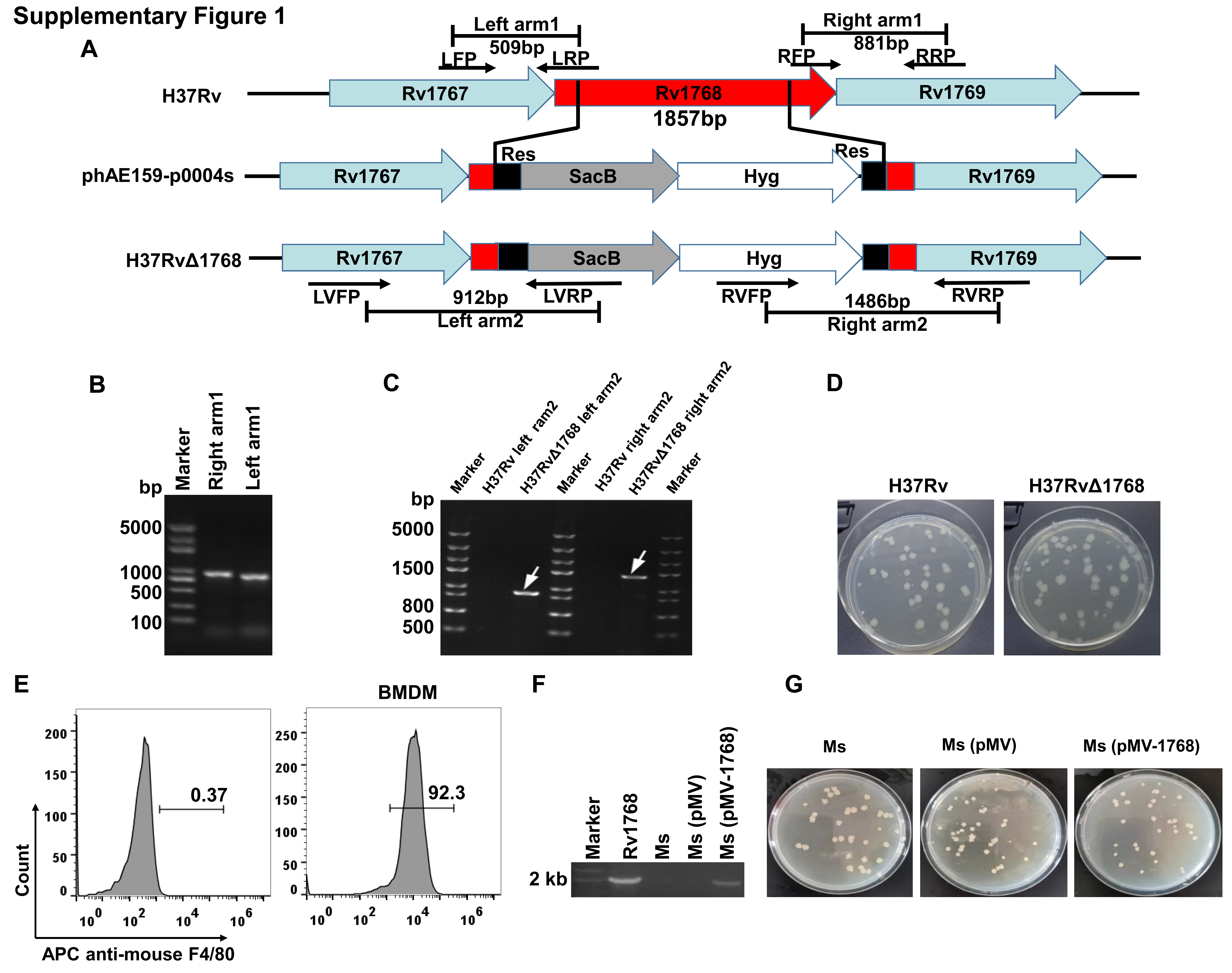

Supplement: FIGURE S1 — Construction of H37RvΔ1768 by phage-mediated allelic exchange. (A) Schematic showing the construction of H37RvΔ1768 strain. LFP, left forward primer; LRP, left reverse primer; RFP, right forward primer; RRP, right reverse primer; LVFP, left verification forward primer; LVRP, left verification reverse primer; RVFP, right verification forward primer; RVRP: right verification reverse primer; Res, resolvase specific site for antibiotic resistance cassette removal. (B) Agarose electrophoresis of the homologous left and right arm. (C) PCR amplification products from H37Rv and H37RvΔ1768. (D) Representative colonial morphology of H37Rv and H37RvΔ1768. (E) Representative flow cytometry analysis of the purity of isolated mouse BMDMs. (F) Agarose electrophoresis of Rv1768 PCR products in recombinant M. smegmatis. (G) Representative colonial morphology of Ms, Ms (pMV) and Ms (pMV-1768). [file Image_1.TIF]

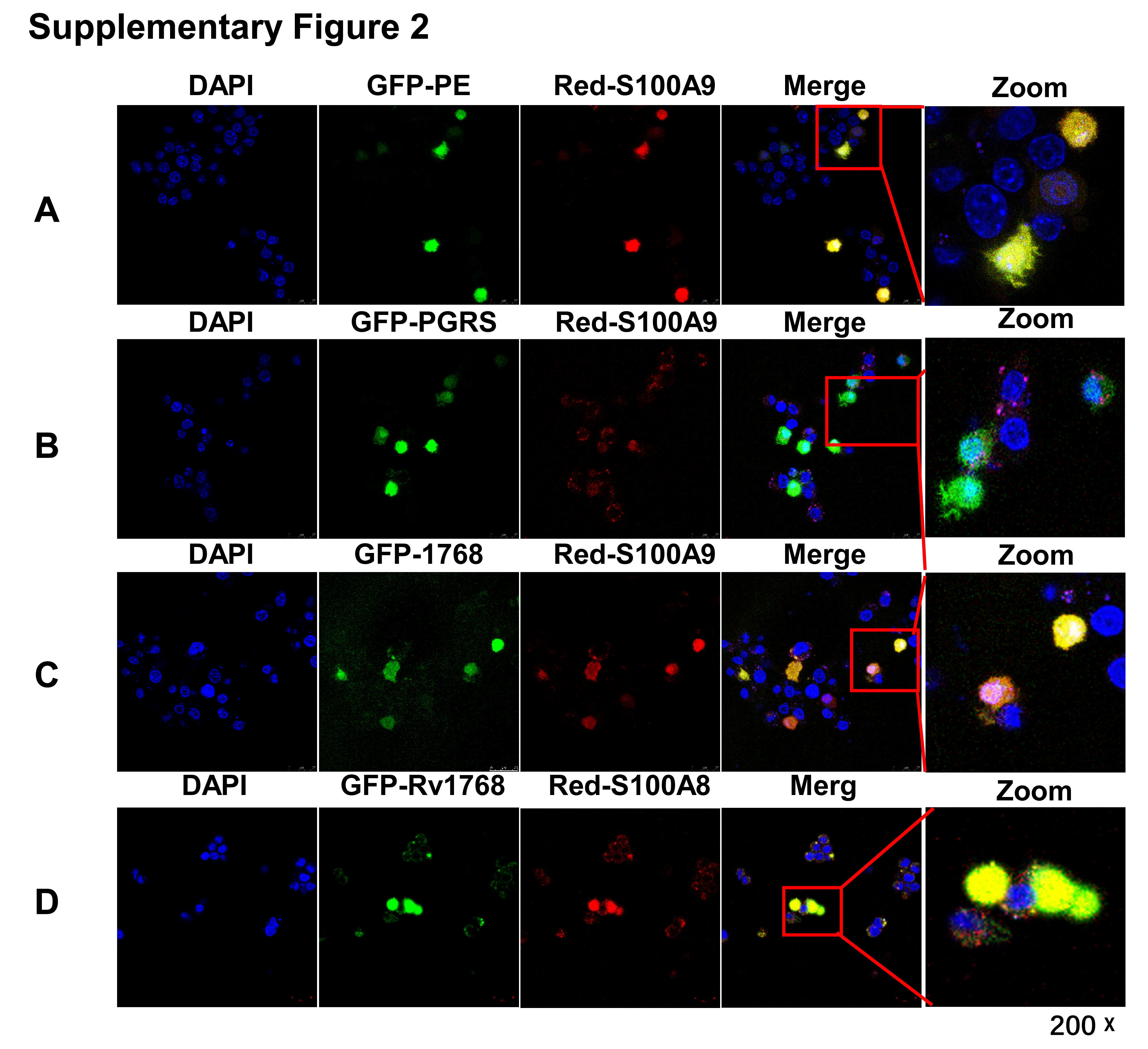

Supplement: FIGURE S2 — Confocal microscope analysis of the co-localization between Rv1768/1768-PE/1768-PGRS and S100A9/S100A8 in RAW264.7 cell. (A–C) Confocal microscopy analysis of co-localization between Rv1768 and S100A9 in RAW264.7 cells. (A) RAW264.7 were co-transfected with pAsRed2-N1-S100A9 and pEGFP-C1-PE, (B) RAW264.7 were co-transfected with pAsRed2-N1-S100A9 and pEGFP-C1-PGRS, (C) RAW264.7 were co-transfected with pAsRed2-N1-S100A9 and pEGFP-C1-Rv1768. At 24 h post transfection, the cellular nuclei were labeled by DAPI. Confocal images were taken with a Leica-LCS-SP8-STED confocal system. (D) Confocal microscopy analysis of the co-localization between Rv1768 and S100A8 in RAW264.7 cells. The cells were co-transfected with pEGFP-Rv1768 and pAsRed2-N1-S100A8. [file Image_2.TIF]

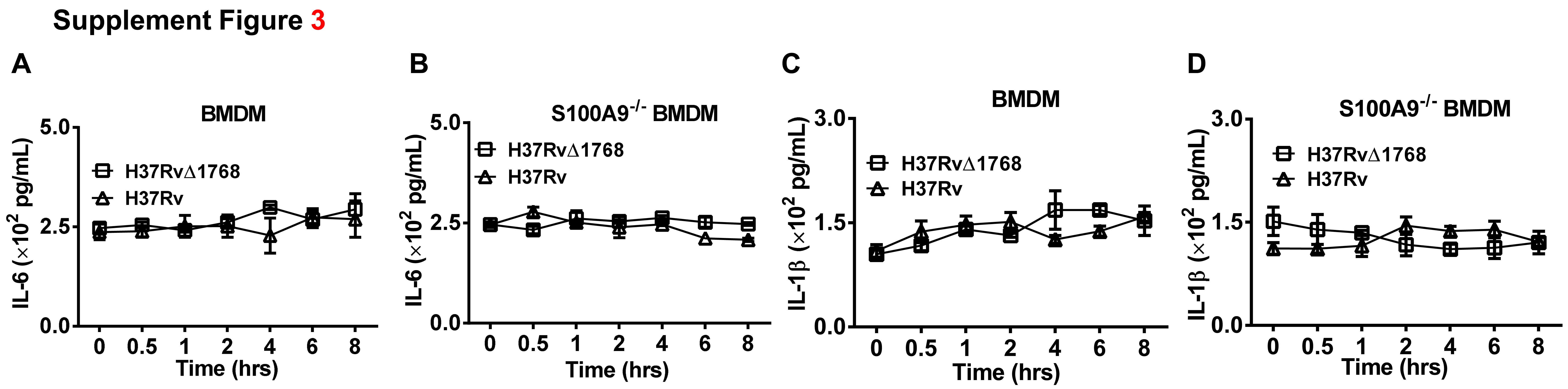

Supplement: FIGURE S3 — IL-6 and IL-1β in the supernatant of macrophages infected with H37Rv or H37RvΔ1768 at different time. (A,B) IL-6 levels in the supernatants of WT (A) and S100A9–/– BMDMs (B) infected with H37Rv or H37RvΔ1768 at MOI = 10 for different time. (C,D) IL-1β levels in the supernatants of WT (C) and S100A9–/– BMDM (D) infected with H37Rv or H37RvΔ1768 at MOI = 10 for different time. Two-way repeated measures ANOVA with Tukey’s post hoc multiple comparison test was used to compare the means across multiple time points and multiple groups. The data are presented as mean ± SD (error bars). Data averaged from at least three independent experiments. *p < 0.05, **p < 0.01, ***p < 0.001, ****p < 0.0001. [file Image_3.TIF]

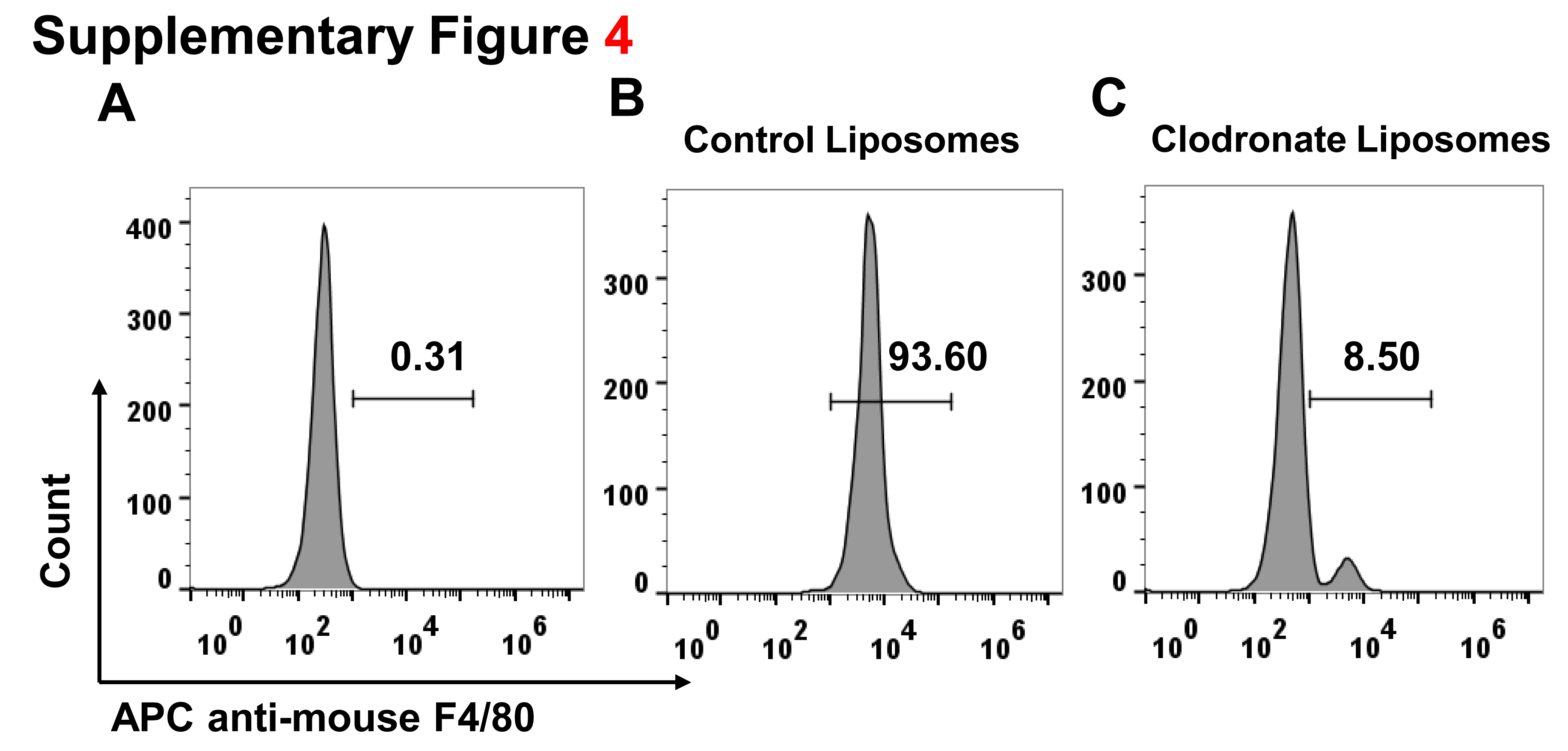

Supplement: FIGURE S4 — FCM analysis for the murine macrophage deal with clodronate liposome (A) Negative control. (B) BMDM from control liposome injected mouse. (C) BMDM from clodronate liposome injected mouse. [file Image_4.TIF]
